# Microevolution of Serial Clinical Isolates of *Cryptococcus neoformans* var. *grubii* and *C. gattii*

**DOI:** 10.1128/mBio.00166-17

**Published:** 2017-03-07

**Authors:** Yuan Chen, Rhys A. Farrer, Charles Giamberardino, Sharadha Sakthikumar, Alexander Jones, Timothy Yang, Jennifer L. Tenor, Omar Wagih, Marelize Van Wyk, Nelesh P. Govender, Thomas G. Mitchell, Anastasia P. Litvintseva, Christina A. Cuomo, John R. Perfect

**Affiliations:** a Division of Infectious Diseases, Department of Medicine, Duke University Medical Center, Durham, North Carolina, USA; b Department of Molecular Genetics and Microbiology, Duke University Medical Center, Durham, North Carolina, USA; c Broad Institute of Massachusetts Institute of Technology and Harvard University, Cambridge, Massachusetts, USA; d European Molecular Biology Laboratory (EMBL), European Bioinformatics Institute, Wellcome Genome Campus, Hinxton, Cambridge, United Kingdom; e National Institute for Communicable Diseases (Centre for Opportunistic, Tropical, and Hospital Infections), A Division of the National Health Laboratory Service, Johannesburg, South Africa; f Mycotic Diseases Branch, Centers for Disease Control and Prevention, Atlanta, Georgia, USA; Institut Pasteur

## Abstract

The pathogenic species of *Cryptococcus* are a major cause of mortality owing to severe infections in immunocompromised as well as immunocompetent individuals. Although antifungal treatment is usually effective, many patients relapse after treatment, and in such cases, comparative analyses of the genomes of incident and relapse isolates may reveal evidence of determinative, microevolutionary changes within the host. Here, we analyzed serial isolates cultured from cerebrospinal fluid specimens of 18 South African patients with recurrent cryptococcal meningitis. The time between collection of the incident isolates and collection of the relapse isolates ranged from 124 days to 290 days, and the analyses revealed that, during this period within the patients, the isolates underwent several genetic and phenotypic changes. Considering the vast genetic diversity of cryptococcal isolates in sub-Saharan Africa, it was not surprising to find that the relapse isolates had acquired different genetic and correlative phenotypic changes. They exhibited various mechanisms for enhancing virulence, such as growth at 39°C, adaptation to stress, and capsule production; a remarkable amplification of *ERG11* at the native and unlinked locus may provide stable resistance to fluconazole. Our data provide a deeper understanding of the microevolution of *Cryptococcus* species under pressure from antifungal chemotherapy and host immune responses. This investigation clearly suggests a promising strategy to identify novel targets for improved diagnosis, therapy, and prognosis.

## IMPORTANCE

Opportunistic infections caused by species of the pathogenic yeast *Cryptococcus* lead to chronic meningoencephalitis and continue to ravage thousands of patients with HIV/AIDS. Despite receiving antifungal treatment, over 10% of patients develop recurrent disease. In this study, we collected isolates of *Cryptococcus* from cerebrospinal fluid specimens of 18 patients at the time of their diagnosis and when they relapsed several months later. We then sequenced and compared the genomic DNAs of each pair of initial and relapse isolates. We also tested the isolates for several key properties related to cryptococcal virulence as well as for their susceptibility to the antifungal drug fluconazole. These analyses revealed that the relapsing isolates manifested multiple genetic and chromosomal changes that affected a variety of genes implicated in the pathogenicity of *Cryptococcus* or resistance to fluconazole. This application of comparative genomics to serial clinical isolates provides a blueprint for identifying the mechanisms whereby pathogenic microbes adapt within patients to prolong disease.

## INTRODUCTION

Pathogenic species of *Cryptococcus* are encapsulated yeasts that normally reside in the environment, where they readily become airborne and can infect humans through inhalation. These yeasts are neurotropic; after causing pulmonary infection, they can spread to the central nervous system (CNS) and cause life-threatening meningoencephalitis. Cryptococcal species have evolved manifold attributes that enable them to progress from latent infection to disease in immunocompetent individuals as well as in patients with suppressed cellular immunity, especially those with HIV/AIDS (1–4). Many of the human risks for increased susceptibility to cryptococcal disease have been determined. With regard to the pathogen, basic research has shown that the *Cryptococcus* genome is dynamic in its plasticity (5), but little is known about the genetic and phenotypic adaptations that the yeast cells undergo to invade, survive, and proliferate in the CNS of patients. To directly examine the properties of cryptococcal cells during this critical process, our previous study profiled the expression of *Cryptococcus* genes at the site of human meningitis. In that study, genes related to catalytic activity and transporters, such as *ENA1* and *CFO1*, were significantly upregulated in cerebrospinal fluid (CSF) during infection, suggesting the importance of such functions for survival of the yeasts in the harsh human environment, specifically within the subarachnoid space (6, 7). Although these studies identified expression signatures during active infection, they provided only an initial glimpse of how this yeast is able to adapt rapidly and continuously to assault by host immune responses and treatment with antifungal drugs.

An independent approach to look at adaptation is to examine mutations that occur over the course of an infection and determine if any confer a selective advantage in the host. Between 10% and 20% of HIV/AIDS patients in South Africa develop recurrent cryptococcal meningitis (8). Consequently, genetic and phenotypic dissection of clinical relapse isolates may elucidate how the initial infecting isolate adapted to the CNS of the host. Microevolution of isolates of *Cryptococcus* has been documented in several cases of recurrent infection as well as by *in vitro* and *in vivo* experiments (9–11). In a recent report of a case of recurrent cryptococcal meningoencephalitis (CM), the genomic sequences of incident and relapse isolates were compared after 77 days in the patient’s CNS (12). Several mutations were identified between these serial isolates, and a frameshift mutation in the AT-rich interaction domain-containing (ARID) transcriptional regulator was implicated in several phenotypic differences, including reduced capsule size and altered carbon source preference. In addition, strains with higher rates of spontaneous mutation may display accelerated microevolution within the host (11), and the serial passage of cryptococcal strains in the same host can change their virulence potential (13). These studies clearly indicated the importance of microevolution in the adaptation of *Cryptococcus* to the human CNS and ability to cause disease.

In an earlier investigation, we examined the incident and relapse isolates of 81 HIV/AIDS patients from South Africa with recurrent cryptococcal meningoencephalitis (14). Each isolate was genotyped by multilocus sequence typing (MLST). As expected from previous studies, the vast majority of cases were caused by isolates of *C. neoformans* var. *grubii*, which consists of three distinct molecular types, VNI, VNII, and VNB (15, 16). Infections with *C. gattii* are less common in South Africa and are caused by molecular type VGI or VGIV (17). After initial diagnosis and treatment with fluconazole (FLZ) and/or amphotericin B (AMB), in most cases, recurrent disease can ensue because of reinfection with a different isolate or persistent infection by the initial isolate and relapse, perhaps due to inadequate antifungal treatment or selection of the more virulent clones within the patient. In this cohort, 89% of relapse episodes were caused by isolates with the same genotype(s) as the incident isolates (14).

To investigate the microevolution of *C. neoformans* var. *grubii* and *C. gattii* in the CNS under selective pressure from the host immune responses and antifungal treatment, we analyzed the incident and relapse isolates from 18 patients. These serial isolates appeared clonal based on MLST analysis (14). Using whole-genome sequencing, we traced the mutational events of each isolate during its passage in the patient and compared these mutations to key phenotypic changes in the same isolates. Correlative changes in genotype and phenotype were detected during *in vivo* microevolution, and multiple genes were implicated in shifts toward resistance to FLZ or adaptation to the host.

## RESULTS

### Whole-genome sequencing of serial isolates.

This investigation included 32 isolates of *C. neoformans* var. *grubii* and six *C. gattii* isolates (see Table S1 in the supplemental material). Each incident isolate was cultured from a specimen of CSF at the diagnosis of cryptococcal meningoencephalitis but prior to treatment. Relapse isolates were obtained from subsequent CSF specimens more than 120 days following collection of the incident isolate.

10.1128/mBio.00166-17.5 TABLE S1  Summary information of all isolates used in this study. (Reproduced from reference 14). Download TABLE S1, PDF file, 0.03 MB. Copyright © 2017 Chen et al. 2017 Chen et al. This content is distributed under the terms of the Creative Commons Attribution 4.0 International license.

**FIG 1:**
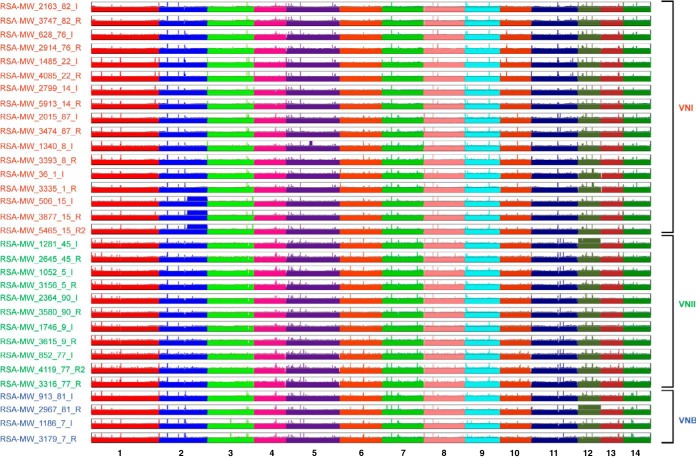
Aneuploid regions of all *C. neoformans* var. *grubii* isolates. For each sequenced isolate, the normalized read depth is shown in 5-kb windows along each chromosome relative to the H99 reference, and average normalized depth on the *y* axis is relative to ploidy levels.

**FIG 2:**
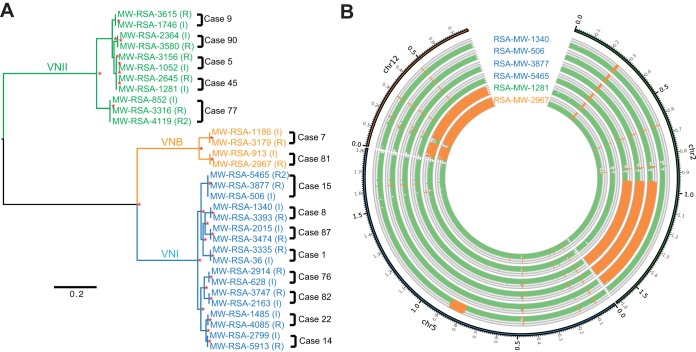
Phylogenetic relationships of the serial isolates and comparison of duplicated genomic regions in six relapse isolates. (A) Phylogenetic tree inferred by using RAxML from 503,457 SNPs in the *C. neoformans* var. *grubii* genome (2.7% of the genome). A total of 1,000 bootstrap replicates were performed, and nodes with 100% bootstrap support are denoted by asterisks (*). (B) Copy number of three *C. neoformans* var. *grubii* chromosomes (chromosomes 2, 5, and 12) for six isolates with a large genomic duplication; orange regions show elevated copy numbers, and regions with normal copy number are shown in green.

Genome-wide variants were identified for each isolate and compared to examine changes during passage. Genomic DNA of each isolate was extracted from a single colony and analyzed by aligning Illumina reads to their most closely related reference assemblies, allowing the identification of single nucleotide polymorphisms (SNPs) and indels (insertion or deletion events; see Materials and Methods). High-depth (268× on average) read alignments covered over 96% of the reference genome nucleotides for most isolates (Fig. 1; see also Fig. S1 and Table S2 in the supplemental material). Stringent filters were applied to SNP calls to reduce false positives, such as near-alignment errors due to adjacent indels, and the final calls were manually validated. To verify the data and check for clonality of serial isolates, a phylogenetic tree of *C. neoformans* var. *grubii* isolates was inferred from the SNPs for each strain (Fig. 2A). This phylogeny confirmed that each patient’s isolates were closely related, suggesting clonality, and concordant with the previous MLST study (14). The patient isolates were comprised of diverse molecular types. The *C. neoformans* var. *grubii* cases included eight with molecular type VNI, five with molecular type VNII, and two with molecular type VNB; the *C. gattii* cases included one with molecular type VGI and two with molecular type VGIV.

10.1128/mBio.00166-17.1 FIG S1  Aneuploid regions of *C. gattii* VGI (A) and VGIV (B) isolates. For each sequenced isolate, the normalized read depth is shown in 5-kb windows along each chromosome relative to the WM276 (VGI) and IND107 (VGIV) references, and the average normalized depth on the *y* axis is relative to the ploidy levels. A large deletion region was identified on chromosome 14 of both isolates from patient 91. Download FIG S1, TIF file, 0.5 MB. Copyright © 2017 Chen et al. 2017 Chen et al. This content is distributed under the terms of the Creative Commons Attribution 4.0 International license.

10.1128/mBio.00166-17.6 TABLE S2  Sequencing coverage, numbers of SNPs, and SRA accession numbers for all sequenced isolates. Download TABLE S2, PDF file, 0.1 MB. Copyright © 2017 Chen et al. 2017 Chen et al. This content is distributed under the terms of the Creative Commons Attribution 4.0 International license.

### SNP calling and comparisons between the serial isolates.

To investigate microevolution during infection, we more closely compared the single nucleotide polymorphism (SNPs) between the isolates from each patient (Table 1). The number of SNPs between incident and relapse pairs ranged from zero (cases 76 and 91) to six (case 1); between the first and second relapse isolates, two and four SNPs were identified (cases 15 and 77, respectively). In total, 43 SNP sites were identified among all the paired *C. neoformans* var. *grubii* isolates. Five SNPs were identified in the two VGIV serial isolates (Table S3), and no SNP was found between the two serial isolates of VGI. Among the *C. neoformans* var. *grubii* isolates, 29 of the 43 SNPs were located within the coding regions of 26 different genes. Remarkably, more than two-thirds (20/29) of these changes resulted in nonsynonymous mutations distributed among 19 genes, and 5 introduced stop codons. For *C. gattii* isolates, three of the five SNPs between VGIV isolates were in coding regions, and two SNPs resulted in nonsynonymous mutations (Table S4). For cases 15 and 77 of *C. neoformans* var. *grubii*, which had second relapse isolates, only half of the SNPs detected between the first and second relapses were retained in the second relapse isolate. Although samples from only two patients were studied here, these findings suggest that perhaps half the mutations that occur during a similar time span of cryptococcal infection are transient or not fixed in the population, which has also been suggested in previous studies in both *Candida albicans* and *Mycobacterium tuberculosis* (18, 19).

**TABLE 1:** Numbers of SNPs, small indels, and large deletions between the incident and relapse isolates or between the first-relapse and second-relapse isolates (case 15R and case 17R)

Case ID^a^	Molecular type	No. of NPs	No. of small indels	No. of large deletions
1	VNI	6	1	0
8	VNI	1	0	1
14	VNI	1	0	0
15	VNI	2	1	0
15R	VNI	2	0	0
22	VNI	1	1	0
76	VNI	0	0	0
82	VNI	1	0	0
87	VNI	4	0	0
7	VNB	2	0	0
81	VNB	5	2	0
5	VNII	1	0	0
9	VNII	2	0	0
45	VNII	4	0	0
77	VNII	6	0	0
77R	VNII	4	0	0
90	VNII	5	0	0
91	VGI	0	0	0
31	VGIV	4	1	0
67	VGIV	1	0	0

a ID, identifier.

10.1128/mBio.00166-17.7 TABLE S3  Detailed information of identified *C. neoformans* var. *grubii* SNPs between the serial isolates. Download TABLE S3, PDF file, 0.04 MB. Copyright © 2017 Chen et al. 2017 Chen et al. This content is distributed under the terms of the Creative Commons Attribution 4.0 International license.

10.1128/mBio.00166-17.8 TABLE S4  Detailed information of identified *C. gattii* VGIV SNPs between the serial isolates. Download TABLE S4, PDF file, 0.02 MB. Copyright © 2017 Chen et al. 2017 Chen et al. This content is distributed under the terms of the Creative Commons Attribution 4.0 International license.

Some of the affected genes are reported to encode potential virulence factors or to be involved in stress responses or are potentially important for fitness and survival within the host. For example, *Crz1* (*Sp1*; CNAG_00156) is a transcription factor activated by calcineurin and regulates cell wall integrity (20). We identified a null mutation of this gene in the incident isolate of case 9 (RSA-MW-2364), and this site reverted in the corresponding relapse isolate (RSA-MW-3580) to produce a functional copy of this gene. In addition to inactivating mutations, nonsynonymous changes were detected in several important genes. Eukaryotic initiation factor 2 alpha (*eIF2A*; CNAG_05366) mediates stress responses, and this function has been conserved across different species (21–24). MCM2 (CNAG_03341) is a member of the minichromosome maintenance (MCM) protein family that is highly conserved throughout evolution and essential for eukaryotic DNA replication (25, 26); in *Saccharomyces cerevisiae*, this gene also responds to ethanol stress (24, 27). The protein kinase *CTK1* (CNAG_04118) was identified in a large-scale screen for genes required for virulence (28).

### Small insertions/deletions (indels) identified in the serial isolates.

In addition to SNPs, indels were detected between incident and relapse isolates. Each candidate indel within a coding region was manually examined for read support, resulting in six short indels verified from five pairs of incident and relapse isolates (Table 1). Each of these six indels caused frame shifts in the corresponding coding regions. Five indels involved the insertion or deletion of one or two nucleotides, and the sixth was a deletion of 25 nucleotides in the relapse isolate. Two of these changes were present only in the initial isolates and reverted in the relapse isolates (from patients 15 and 81) compared to the reference genome (Table 2). Among these genes, the gene encoding the eIF2A kinase, *GCN2* (CNAG_06174), may be involved in several stress responses (29, 30), and the *RPD3* (CNAG_05690) gene encodes histone deacetylase and is a repressor that regulates a large number of genes (31). A frameshift due to the insertion of a single base was detected in *MLH3* (CNAG_01037), encoding an endonuclease involved in DNA mismatch repair (32) that was recently identified in a screen for genes required for *C. neoformans* var. *grubii* virulence in three experimental infection models (33).

**TABLE 2:** Comparison of small indels that differed between paired serial isolates^a^

CHR	POS	Reference (H99) nucleotide(s)	ALT	Indel result for case:					Gene ID	Name	Description
				1	15	22	81	87			
5	1378568	C	CT					0–1	CNAG_01037	*MLH3*	DNA mismatch repair protein MLH3
7	623649	TC	T			0–1			CNAG_05690	*RPD304*	Histone deacetylase RPD3
10	405507	GCTTCCGCTTCCGCTGCACGACCGAGT	G				0–1		CNAG_04786		Hypothetical protein
12	126473	AT	A	0–1^a^					CNAG_06033		*pfkB* family carbohydrate kinase superfamily
12	520033	CCT	C				1–0		CNAG_06174	*GCN2*	Pek/GCN2 protein kinase
13	195919	TC	T		1–0				CNAG_06325	*BYR4*	GTPase activator

a “0–1” and “1–0” denote the gain and loss, respectively, of a small indel between initial and relapse isolates of the indicated cases. “0” represents a base(s) identical to that in the reference genome (H99), and “1” indicates the alternative (ALT) sequence. CHR, chromosome; POS, position.

### Variation in copy number was discovered in several genes and genomic regions.

In addition to SNPs and small indels, the comparison of genomic sequences between serial isolates revealed large stretches that differed in copy number. The relapse isolate of patient 8 (RSA-MW-3393) acquired a large deletion of a region on chromosome 3 (chr3) from nucleotide 1303709 to nucleotide 1319672 (~16 kb). Six genes are located in this region, including an oxygenase (CNAG_02580), Cas33 lipase (CNAG_02581), a methyltransferase (CNAG_02583), and an ARID domain protein (CNAG_02579). A recent study of one pair of serial isolates found a single mutation of the ARID domain protein that may contribute to multiple phenotypic changes (12). Another large duplication region was observed on chromosome 5 from nucleotide 791401 to nucleotide 876600 (85.2 kb) of the incident isolate from patient 8 (RSA-MW-1340), with 28 genes duplicated in this region (Fig. 2B; Table 3). Of these genes, deletions of *SET101* (CNAG_01243) and *UFD4* (CNAG_01251) were shown to attenuate the virulence of *C. neoformans* var. *grubii* in mice; *SET101* was also related to melanin production (28). *CDA2* (CNAG_01230) and *CDA3* (CNAG_01239) encode two of the three deacetylases that account for the production of chitosan, which is necessary for maintenance of the cryptococcal cell wall and capsule (34). *PMC1* (CNAG_01232) encodes a calcium transporter, which provides Ca^2+^ tolerance and is essential for the progression of pulmonary infection in mice and dissemination to the brain (35). *HapX* (CNAG_01242) regulates iron acquisition and metabolism (36), and a recent study of the other iron acquisition gene, *FRE3*, showed that this type of gene can be viewed as a “virulence adaptation gene” and evolved in mammalian hosts (37).

**TABLE 3:** Genes that differed in copy number between serial isolates^a^

Gene ID	Gene name	Description of product	CHR	Case 8	Case 22	Case 5	Case 77	Case 81	Case 82	Case 9
CNAG_02579		ARID domain protein	3	1.00 → 0						
CNAG_02580		2OG-Fe(II) oxygenase superfamily domain	3	1.00 → 0						
CNAG_02581	*CAS33*	CAS33 capsule-associated protein	3	1.00 → 0						
CNAG_02582		Hypothetical protein	3	1.00 → 0						
CNAG_02583		tRNA (guanine10-N2)-methyltransferase	3	1.00 → 0						
CNAG_02584		Serine/threonine-protein phosphatase 2A activator 1	3	1.00 → 0						
CNAG_07981		Hypothetical protein	5	1.91 → 1.00						
CNAG_01229		l-Mandelate dehydrogenase	5	1.91 → 1.00						
CNAG_01230	*Cda2*	Chitin deacetylase	5	1.91 → 1.00						
CNAG_01231		Agmatinase	5	1.91 → 1.00						
CNAG_01232	*PMC1*	Putative calcium-transporting ATPase	5	1.91 → 1.00						
CNAG_01233		Hypothetical protein	5	1.91 → 1.00						
CNAG_01234		Spore wall assembly-associated protein	5	1.91 → 1.00						
CNAG_01235		DNA-directed RNA polymerase II subunit RPB2	5	1.91 → 1.00						
CNAG_01236		rRNA-processing protein EBP2	5	1.91 → 1.00						
CNAG_01237		Hypothetical protein	5	1.91 → 1.00						
CNAG_01238		Arginine biosynthesis ArgJ, mitochondrial	5	1.91 → 1.00						
CNAG_01239	*Cda3*	Chitin deacetylase	5	1.91 → 1.00						
CNAG_01240		Hypothetical protein	5	1.91 → 1.00						
CNAG_01241		Enzyme regulator	5	1.91 → 1.00						
CNAG_01242	*HapX*	bZIP transcription factor	5	1.91 → 1.00						
CNAG_01243	*SET101*	Histone-lysine *N*-methyltransferase, H3 lysine-4 specific	5	1.91 → 1.00						
CNAG_01244		Hypothetical protein, hypothetical protein, variant	5	1.91 → 1.00						
CNAG_01245		Hypothetical protein	5	1.91 → 1.00						
CNAG_01246		Hypothetical protein	5	1.91 → 1.00						
CNAG_01247		Hypothetical protein	5	1.91 → 1.00						
CNAG_01248		Vacuole morphology and inheritance protein 14	5	1.91 → 1.00						
CNAG_01249		Hypothetical protein	5	1.91 → 1.00						
CNAG_01250		tRNA ligase	5	1.91 → 1.00						
CNAG_01251	*UFD4*	e3 ubiquitin-protein ligase	5	1.91 → 1.00						
CNAG_01252		Thiosulfate/3-mercaptopyruvate sulfurtransferase	5	1.91 → 1.00						
CNAG_01253		Mitochondrial import inner membrane translocase subunit TIM10	5	1.91 → 1.00						
CNAG_01254		Protein-tyrosine-phosphatase	5	1.91 → 1.00						
CNAG_01255		Hypothetical protein	5	1.91 → 1.00						
CNAG_07876		Hypothetical protein	14		1.00 → 1.90	1.00 → 2.00	1.96 → 1.00	1.00 → 1.86	1.84 → 1.00	
CNAG_01370		Hypothetical protein	5	1.00 → 2.00						
CNAG_01371	*CRG2*	Regulator of G protein signaling	5	1.00 → 2.00						
CNAG_00039		Hypothetical protein	1							1.00 → 12.99
CNAG_07308		Hypothetical protein	1							2.06 → 12.99
CNAG_00040	*ERG11*	Lanosterol 14-alpha-demethylase	1							2.06 → 12.99

a In the columns below each case number, the first number indicates the number of copies of the listed gene in the initial isolate, and the second number indicates the number of copies in the relapse isolate.

By closer examination of the genes within the deletion and duplication genomic regions, we detected six genes with more than two copies in the relapse isolates of four patients (Table 3). Four of the six genes were functionally unknown, and the other two genes were *ERG11* (CNAG_00040) and *CRG2* (CNAG_01371). *ERG11* encodes the FLZ target enzyme, sterol 14α-demethylase (38). Crg2 is a regulator of G-protein signaling homologs, and the null mutant of *CRG2* has been shown to attenuate virulence in mice (39). In the relapse isolate from patient 9, the copy number of a small region of chromosome 1 that includes three genes (CNAG_00039, *ERG11*, and CNAG_07308) was dramatically increased. Two copies were present in the incident isolate (RSA-MW-1746) and 13 copies in the relapse isolate (RSA-MW-3615). This remarkable amplification of *ERG11* was likely selected following treatment of this patient with FLZ.

The apparent plasticity of the cryptococcal genome allows frequent duplication of chromosomes *in vivo* (40, 41). In our study, we identified a partial duplication of chromosome 2 from around nucleotide 945000 to the end of the chromosome in all three isolates from patient 15 and duplication of chromosome 12 in the incident isolate from patient 45 and the relapse isolate from patient 81 (Fig. 2B). Neither chromosome 2 nor chromosome 12 duplications is known to confer increased drug resistance; however, they may provide other selective advantages or may simply result from genomic instability during infection.

### Higher resistance to fluconazole in the relapse isolates.

Since 16 of the 18 patients in this study were treated with antifungal drugs (Table S1), we sought to examine the level of drug resistance in each relapse isolate. Two patients, cases 45 and 90, lacked a record of receiving antifungal treatment (14). Three patients (cases 1, 22, and 90) were treated with both AMB and FLZ, and seven patients received either AMB or FLZ (Table S1). By the use of the CLSI broth microdilution method to determine MICs, each isolate was tested for susceptibility to FLZ. In total, five relapse isolates had 4-fold or greater FLZ MICs than the corresponding incident isolates (Table 4 and S6). Among all the isolates, the relapse isolate (RSA-MW-3615) from patient 9 had the highest FLZ MIC (128 μg/ml), 16-fold greater than the MIC seen with the incident isolate (RSA-MW-1746; MIC, 8 μg/ml). Two genes, *BYR4* (CNAG_06325) and *ERG11*, were associated with increased resistance to FLZ in case 15 and case 9, respectively. A single base deletion was identified in *BYR4* for the incident isolate from patient 15, but this change reverted in the relapse isolate (Table 2). Byr4 is a dosage-dependent regulator of cytokinesis (42), and it has been reported to be essential for resistance of *C. neoformans* var. *grubii* to FLZ (43). The deletion of the nucleotide caused a frameshift affecting two-thirds of the protein length, likely to produce a loss of Bry4 function. Therefore, the changes in FLZ resistance in case 15 serial isolates might be directly associated with this mutation.

10.1128/mBio.00166-17.10 TABLE S6  Summary information of all phenotypic tests. Download TABLE S6, PDF file, 0.04 MB. Copyright © 2017 Chen et al. 2017 Chen et al. This content is distributed under the terms of the Creative Commons Attribution 4.0 International license.

**TABLE 4:** Serial isolates with more than two significant phenotypic changes (more than 4× FLZ resistance changes or other tests with *P* value less than 0.05)

Phenotypic test	Type	Phenotypic test results for case:						
		7	8	9	15	45	77	81
MIC (µg/ml)	Incident	1	4	8	2	1	2	4
	Relapse	16	8	128	8	8	4	64
37°C YPD	Incident	1.739^a^	0.748	1.614	1.736	1.515	1.535	1.744
	Relapse	1.859	1.854	1.332	1.857	1.543	1.715	1.832
	*P* value		0.015					
39°C YPD	Incident	0.604	0	1.673	1.738	0.141	0.459	1.713
	Relapse	0	1.015	0.043	1.702	0.518	1.820	0.592
	*P* value		<0.001	0.035			0.011	0.004
YPD + 0.03% SDS	Incident	1.810	0.671	1.885	0	3.413	0	2.350
	Relapse	2.069	1.693	1.837	1.702	3.324	2.197	2.642
	*P* value				0.007		0.004	
YPD + 0.5 g/liter caffeine	Incident	1.077	1.261	0.970	1.997	1.516	0.014	1.216
	Relapse	1.158	1.535	0.872	1.398	0.911	1.069	2.836
	*P* value						0.003	
YPD + 100 mg/liter l-DOPA	Incident	0.278	0.245	0.173	0.364	0.546	0.468	0.152
	Relapse	0.271	0.478	0.184	0.292	0.471	0.475	0.318
	*P* value				0.045			0.037

a The normalized mean values (other than MIC values and *P* values) from three independent tests (see Materials and Methods).

**FIG 3:**
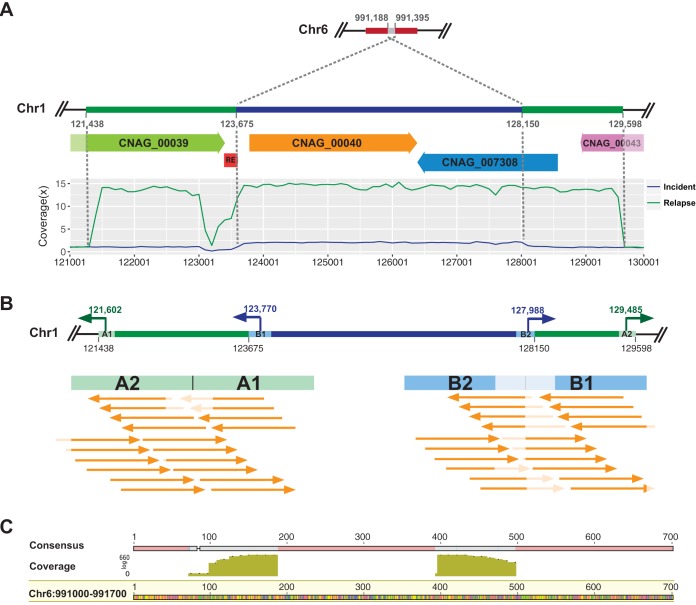
Analyses of the highly amplified genomic sequences containing *ERG11* in the isolates from patient 9. (A) The region of chromosome 1 (Chr1) that includes the ERG11 gene (CNAG_00040) was duplicated on Chr6. Two copies of this region on Chr1 from nucleotide 123675 to nucleotide 128150 were identified in the incident isolate (RSA-MW-1746), and the extra copy was mapped as an insertion into Chr6. A larger duplicated region was identified in the relapse isolate (RSA-MW-3615) with an average of 13× coverage on chromosome 1 from nucleotide 121438 to nucleotide 129598. (B) The TagMap assay was used to analyze the duplicated regions. Primers were designed within 200 bp of the break points. The two flanking regions were concatenated in reverse order (A2 → A1 and B2 → B1), and the sequencing reads were aligned to verify their tandem duplication. A large proportion of the reads could be aligned to the A2–A1 junction, indicating tandem alignment of the duplicated region in RSA-MW-3615. None of the reads aligned to the B2–B1 junction, indicating that no tandem arrangement of the duplicated region had occurred in RSA-MW-1746. These results provide more evidence that the other copy was inserted on Chr6. (C) Sequencing reads from the TagMap assay of RSA-MW-1746 aligned to Chr6 confirmed the insertion of an additional copy of this region from 991,188 to 991,395.

The duplication of genes and genomic regions is another well-documented mechanism for developing resistance to FLZ. In fact, heteroresistance in *Cryptococcus* has been described and shown to be due to aneuploidy, with duplication of chromosome 1 containing the gene (AFR1) encoding an efflux pump and the target (*ERG11*) of FLZ (44). This duplication can be observed *in vivo* when the yeast is under stress, but the extra copy is lost under nonstress growth conditions (40). Therefore, our sequencing of a single colony after many passages *in vitro* may have missed some changes in chromosomal aneuploidy. Furthermore, we observed no changes or duplication in the *AFR1* gene between any paired isolates. In case 9, an alternative yeast strategy of resistance was used. The increased MIC of the relapse isolate can be attributed to the extensive local duplication of *ERG11* and elevated production of Erg11, the target of FLZ. Indeed, the copy number of this small genomic region increased from 2 in the incident isolate to 13 in the relapse isolate (Table 3). By more finely mapping the read coverage boundaries of the duplicated regions in the two genomes, we found that the duplicated region on chromosome 1 of the incident isolate (from nucleotide 123657 to nucleotide 128150) was smaller than in the relapse isolate (from nucleotide 121438 to nucleotide 129598), suggesting that multiple independent amplifications can contribute to resistance (Fig. 3A). Previous studies reported that increasing the copy number of *ERG11* or the related region can produce resistance to FLZ in *C. neoformans* var. *grubii* (40). However, to the best of our knowledge, this is the first observation that *C. neoformans* var. *grubii* can so markedly amplify *ERG11*.

### Investigating mechanisms of *ERG11* amplification.

To investigate the underlying duplication mechanism of this small region, we mapped the surrounding regions using a Tagmentation-based mapping (TagMap) assay (45) on both isolates of case 9. Specific primers were designed adjacent to the break points of the duplications (Fig. 3B), and the duplicated and flanking sequences were amplified and sequenced (Materials and Methods). This sequencing mapped the two copies of this region in RSA-MW-1746 to two different chromosomes. One copy was found on chromosome 1, the typical locus in all isolates, and the second copy was located on chromosome 6, replacing the original sequence from nucleotide 991188 to nucleotide 991395 in the 3′-to-5′ direction compared to the H99 reference genome (Fig. 3C). The same repetitive element was located near the 5′ end in both copies, suggesting that the duplication of this region in RSA-MW-1746 could have been mediated by this element. In relapse isolate RSA-MW-3615, the extra copy on chromosome six was retained as in the incident isolate, but a tandem duplication of a larger region was observed on chromosome 1 which created another 11 extra copies of *ERG11* and the two other genes. As noted, amplification of this small region correlated with the significant increase of FLZ resistance, and sequencing revealed the precision and efficiency of this change.

### Mutations may explain virulence changes.

**FIG 4:**
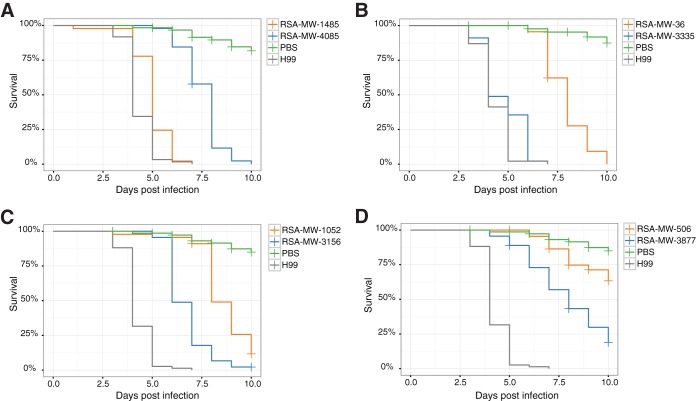
Survival curves for *Galleria mellonella* infected with incident and relapse isolates from patients 22 (A), 1 (B), 5 (C), and 15 (D). H99 (gray) and phosphate-buffered saline (PBS) (green) were used as positive and negative controls in the experiments. Fifteen larvae were infected per isolate, and each isolate was evaluated three times. Mantel-Cox tests showed significant differences (*P* < 0.01) in virulence between the incident (orange) and relapse (blue) isolates in these samples.

The relative pathogenicities of the serial isolates were experimentally compared *in vivo* using larvae of *Galleria mellonella*. For each test, 15 larvae were infected with a comparable inoculum, and mortality was recorded over a period of 10 days; each isolate was tested independently at least three times (Materials and Methods) (Table S5). In 4 (22.2%) of the 18 cases, the incident and relapse isolates significantly differed in this measure of virulence (Gehan-Breslow-Wilcoxon test, *P* value < 0.01). The relapse isolates from patients 1, 5, and 15 were significantly more virulent than the corresponding incident isolates. In contrast, the relapse isolate from patient 22 was less virulent in this assay than the incident isolate (Fig. 4).

10.1128/mBio.00166-17.9 TABLE S5  Summary information of *Galleria mellonella* injection tests. Download TABLE S5, PDF file, 0.1 MB. Copyright © 2017 Chen et al. 2017 Chen et al. This content is distributed under the terms of the Creative Commons Attribution 4.0 International license.

**FIG 5:**
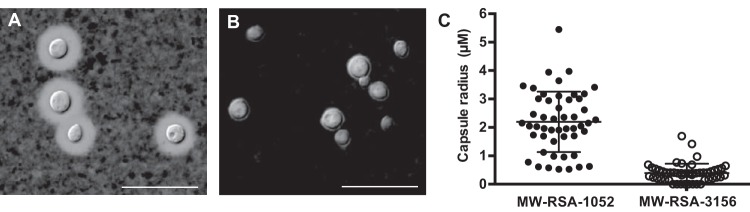
(A and B) Variations in capsule size were observed between the initial isolate, MW-RSA-1052 (A), and the relapse isolate, MW-RSA-3156 (B). Isolates were streaked onto YPD plates and grown for 3 days at 30°C, a condition that typically results in limited capsule production. An individual colony was resuspended in PBS containing India ink, subjected to brief vortex mixing, and examined at ×63 magnification. Bars, 20 µM. (C) The capsule radius for 50 cells was measured. The incident isolate had a much larger capsule size than the relapse isolate. *P* value, <0.0001. The cell body sizes were similar (data not shown).

Examining the SNP differences between the isolates that differed in virulence revealed that notable genes are affected. We identified an interesting SNP between the two serial isolates of patient 5 which was located in the intron of *PKR1* (CNAG_00570). This mutation changed the acceptor site of the first intron of *PKR1* in the incident isolate from AG to CG (on the reverse strand) (Table S3); the high conservation of splice acceptor sites in *Cryptococcus* (5) suggests that this change would cause a splicing error in *PKR1*. Previous studies have reported that the *PKR1* gene encodes the regulatory subunit of protein kinase A (PKA) and that *pkr1* mutant strains overproduce their capsules and are hypervirulent (46, 47). Therefore, we examined capsular phenotypes of the two paired isolates to determine if there were any phenotypic consequences of the SNP. As displayed in Fig. 5, the incident isolate (MW-RSA-1052) with the mutation produced substantially more capsule than the relapse isolate (MW-RSA-3156), and this observation supports the concept of a potential dysfunction of PKR1 between these isolates. For instance, this may suggest that the splicing site mutation in *PKR1* might lead to a defect in removing this intron and/or it may be alternatively spliced. However, we are not clear about the mechanism for the more virulent phenotype of the smaller capsuled relapse isolate in our *in vivo* test. In patient 15, we hypothesize that the attenuated virulence of the incident isolate might also be related to the mutated *BYR4*. Furthermore, this gene has been reported to be essential for viability in *Schizosaccharomyces pombe* (42, 48). In the relapse isolate from patient 1, we identified a single nucleotide deletion causing a frameshift on CNAG_06033 and nonsynonymous substitutions on CNAG_07362 (Table 2 and Table S3). However, the functions of these two genes are yet to be determined. Finally, the relapse isolate from patient 22 had a deletion that caused a frameshift in the *RPD304* (CNAG_05690) gene. *RPD304* is a class II histone deacetylase, and the disruption of this gene has been shown to reduce greatly the virulence of *C. neoformans* var. *grubii* in mice (28), which may explain the attenuated virulence of this relapse isolate (Fig. 4).

### Characterization of additional phenotypic changes in the serial isolates.

To investigate whether the microevolutionary genetic alterations produced phenotypic changes besides FLZ resistance that might be related to virulence and/or host adaptation, we examined several other clinically relevant phenotypes: the ability to survive and proliferate at temperatures of 37°C and 39°C or in the presence of SDS or caffeine, which may perturb cell wall integrity, and the production of melanin, a crucial virulence factor. We developed a high-throughput phenotypic testing method using a BM5 robot and SGAtools (49) that can analyze up to 64 isolates on one plate (Fig. S2) (see Materials and Methods). The results showed that some serial isolates had variable responses to the stress conditions (Table S6). The isolates from patients 8, 15, and 81 had significant phenotypic differences under two or more sets of conditions (Table 4). The significantly lower resistance to SDS of the incident isolate from patient 15 may also be related to the mutation in the *BYR4* gene in it, which is essential for cell wall formation in *S. cerevisiae* (50).

10.1128/mBio.00166-17.2 FIG S2  Phenotypic assays of serial isolates. (A) In each test, a BM5 robot was used to create 24 clones of every isolate, and each set of 24 clones is denoted by a different color on the plate. Each plate can accommodate up to 64 isolates. (B) Each test included two replicates of up to 24 isolate clones, and at least three independent tests were performed for each set of conditions. (C) Images of each plate were taken using the same settings and analyzed using SGATools (1). Shadow areas indicate that no samples were plotted in these regions. The upper left image is representative of the assay for melanin; the lower right image depicts a representative test for growth. Download FIG S2, EPS file, 9.1 MB. Copyright © 2017 Chen et al. 2017 Chen et al. This content is distributed under the terms of the Creative Commons Attribution 4.0 International license.

The relapse isolate from patient 8 has significantly higher temperature tolerance at both 37°C and 39°C (Table 4). The genetic differences between serial isolates from patient 8 include a large deletion region (16 kb) on chr3 of the relapse isolate (RSA-MW-3393). Three of these six genes lost in this region have reported roles in stress responses and pathogenesis. CNAG_02584 is a homolog of *S. cerevisiae* genes *RRD1* and *RRD2*, which are putative activators of the phosphotyrosyl phosphatase gene, PP2A. PP2A is implicated in cell growth and signaling, and phosphorylation of PP2A is required for the stress response in *S. cerevisiae* and other eukaryotes (51, 52). Moreover, a previous study showed that deletion of *RRD1* and *RRD2* can enhance the resistance of *S. cerevisiae* to caffeine (53). Cas33 (CNAG_02581) is involved in formation of the cryptococcal capsule (54), the major virulence factor of pathogenic *Cryptococcus* species (55). CNAG_02583 encodes a tRNA (guanine 10-N2) methyltransferase; methylation of tRNA has been linked to protein synthesis, infections, temperature, and immune responses in several systems (56, 57). The ARID domain protein (CNAG_02579), also in this deleted region, was previously shown to be prematurely truncated by a frameshift mutation in a relapse isolate (12).

In case 81, the relapse isolate had reduced growth at 39°C in comparison to its corresponding incident isolate but produced more melanin (Table 4). Comparing the genetic differences between these two isolates, a small indel in *GCN2* (CNAG_06174) was identified in the incident isolate (RSA-MW-913) but was reverted in the relapse isolate (Table 2). While GCN2 is involved in responses to amino acid starvation, intercellular acid stress, and oxidative stress response in *S. cerevisiae* (58–60), a melanin synthesis-related gene, *Dct*, is also regulated by GCN2 (30). Thus, the difference in melanin production may be explained by the presence of the indel in *GCN2*.

## DISCUSSION

Microbes have developed fascinating genetic plasticity to adapt to a variety of stress conditions within a very short time through mechanisms of microevolution. Understanding these processes and the magnitude of their influence on host adaptability and drug resistance may highlight targets for new strategies and/or drugs to treat infections. However, most of the previous analyses on microevolution have been performed with *in vitro* systems and animal models (5, 61). Here, the complete genome comparisons of clonal serial isolates from relapsing patients provided a specific window to investigate the microevolution of *Cryptococcus* within the human subarachnoid space, primarily under conditions of drug treatment. In these longitudinal samples, the shortest time period between the collection of the incident isolate and collection of the relapse isolate was 124 days for our paired cases, and most isolates had a much longer exposure to the human CNS. We estimate that the mutation rate in the members of this cohort averaged approximately one polymorphism every 58 days. This rate could have been higher in other isolates such as those with mutations in mismatch repair genes (*SGF29* and *MSH2*), which have been identified in clinical isolates (62). In our study, we identified a frameshift mutation in *MLH3*, which is involved in a particular class of mismatch repair of frameshift mutations in *S. cerevisiae* (63), but this change did not appear to increase the observed mutation rate.

To study the microevolution of these isolates, we compared genetic and phenotypic changes for all the paired isolates. High-coverage whole-genome sequencing data were obtained for each isolate, and SNP and indel calling pipelines were optimized to reduce false positives. In parallel, we established highly reproducible assays to evaluate clinically significant phenotypes in a large number of isolates. The combination of these two approaches provided an efficient and reliable strategy to suggest associated phenotypic and genetic changes.

The analyses of serial isolates from 18 patients revealed that only a few genetic changes between incident and relapse isolates were associated with prolonged disease. The low number of mutations is consistent with a previous report of a single case of *C. neoformans* var. *grubii* serial isolates (64). Indeed, more than 40% of the SNPs between serial isolates of these treated patients produced nonsynonymous mutations, which suggests the functional selection of genetic changes that promoted survival and proliferation of the cryptococcal cells. This supposition was supported by the finding that several genetic alterations in the relapse isolates led to phenotypic changes likely to enhance host adaptation or resistance to treatment. For example, the relapse isolate of patient 9 was found to have multiple duplications of a small genomic region carrying *ERG11*, the target gene of FLZ, and this increase in gene copy number was associated with the acquisition of a dramatic increase in resistance to FLZ. The acquisition of tandem duplications of *ERG11* may provide drug resistance that is more stable than that of isolates that acquire chromosome 1 aneuploidies, known to contribute to heteroresistance. A different deletion, in patient 15, caused a frameshift mutation of the *BYR4* gene in the incident isolate, and this genetic change provides an explanation for the increased resistance to FLZ in the relapse isolate. Among the patients in this investigation, 10 were known to have received FLZ, albeit with different regimens, and in 5 cases, the relapse isolates had 4-fold or greater increases in FLZ MICs (Table 4).

Apart from resistance to FLZ, five other clinically relevant phenotypes were evaluated and compared among the isolates. Among the other phenotypes that we compared, a minority of patient isolates differed in growth at higher temperature, response to chemical stress, or melanin production. Virulence was tested with the standard model of infecting and quantifying the survival of wax moth larvae. The paired isolates from four patients differed significantly in virulence; in three cases, the relapse isolate was more lethal than the incident isolate, and in the fourth case, it was less virulent than the incident isolate (Fig. 4). Few of the genetic alterations in these isolates were linked to the genes that could account for these differences. However, the results are not unexpected in that numerous studies have confirmed that cryptococcal pathogenicity is polygenic and that the genes required for growth in the mammalian CNS may not be essential for larval lethality. In the subarachnoid space, cryptococcal cells are under constant stress. Even in the absence of mutations or genomic rearrangements, the expression of critical genes may be regulated as needed. Nevertheless, as detailed in Results, the paired isolates from all four cases had indels or SNPs that altered genes reported to affect cryptococcal virulence, such as *PKR1* and *RPD304*.

In this study, we tried to link phenotypic changes to genetic changes to reveal the contribution of microevolution in building pathogenicity of a microorganism within the human body. Clearly, we successfully identified some mutations that were very likely selected for enhancement of antifungal drug resistance or other stress conditions, but we also found that strong phenotypic changes were observed between the paired isolates in several samples but that no or very limited genetic changes were identified. We speculate that there are three major possible reasons for the results. First, the genome sequencing method that we used was not able to detect the epigenetic modifications between the serial isolates, which have been shown to affect host-pathogen interactions in *C. albicans* and other pathogens (65–67). The modulation of gene expression through histone deacetylation or nucleotide methylation has also been implicated in affecting the fitness and pathogenicity of *C. neoformans* var. *grubii* (68, 69). A recent comparison of closely related strains of *C. gattii* with different phenotypes and genetic changes found mutations in two histone deacetylases (70). The second reason that is the general method used in SNP calling from the next-generation sequencing (NGS) data usually has around a 1% error rate (71, 72); consequently, we applied very rigorous settings in variant calling and followed that by a manual check to ensure the accuracy of the result. Thus, an increase in the false-negative rate may be inevitable. Third, reference genome-based analysis may miss genes unique to isolates from the other lineages. For example, our *C. neoformans* var. *grubii* reference genome that we used was H99, which is a representative of the VNI lineage, and there are two other lineages, VNII and VNB, in *C. neoformans* var. *grubii* (73).

In all, approximately a quarter of strains changed their stress and virulence phenotypes within 3 to 4 months of human subarachnoid space exposure. While genetic changes of *Cryptococcus* occur on a real-time basis during human CNS infections, additional work will be needed to study the impact of specific changes to more directly link them to a phenotype. The adaptation of *Cryptococcus* to the host response and treatment may further highlight genes that are critical for infection and help to understand the dynamics of infection that are important to consider in developing new interventions.

## MATERIALS AND METHODS

### DNA isolation and sequencing.

From a previous investigation of HIV/AIDS patients who relapsed with CM (14), we selected serial isolates from 15 cases of CM caused by *C. neoformans* var. *grubii* (8 of molecular type VNI, 2 VNB, and 5 VNI) and 3 cases due to *C. gattii* (1 VGI and 2 VGIV) (Table 1). From these cases, we analyzed all 38 serial isolates (17 of molecular type VNI, 4 VNB, 11 VNII, 2 VGI, and 4 VGIV). Each isolate was recovered from a freezer stock, thawed, streak-plated on yeast extract-peptone-dextrose (YPD) agar, and grown at 37°C for 36 to 48 h to produce isolated colonies. A single colony was streaked to a fresh YPD plate and grown for 24 h, a portion of the yeast cells (~100 μl) was used for DNA isolation with a MasterPure yeast DNA purification kit (Epicenter, Madison, WI) from sheared genomic DNA of each isolate as directed by the manufacturer’s instructions, and a small insertion library was constructed and sequenced on an Illumina HiSeq 2000 sequencer, generating between 14 and 150 million 101-bp paired-end reads per isolate. This resulted in a 56-fold to 603-fold average depth of aligned bases compared to *C. neoformans* var. *grubii* H99 genome or the known sequenced *C. gattii* genomes.

### Read alignment and variant identification.

Illumina reads were aligned to one of the reference genome assemblies using Burrows-Wheeler Aligner (BWA) v0.7.4-r385 mem (76) and converted to sorted BAM format using SAMtools v0.1.9 (r783) (77). The Genome Analysis Toolkit (GATK) v2.7-4-g6f46d11 (78) was used to call both variant and reference nucleotides from the alignments. Briefly, the Picard tools *AddOrReplaceReadGroups*, *MarkDuplicates*, *CreateSequenceDictionary*, and *ReorderSam* were used to preprocess the alignments (http://broadinstitute.github.io/picard/). We then used GATK RealignerTargetCreator and IndelRealigner to resolve misaligned reads close to indels on serial isolate pairs to avoid discrepancies between isolates. Next, GATK Unified Genotyper (with the haploid Genotyper ploidy setting) was run with both SNP and indel genotype likelihood models (GLM). We also ran Base Recalibrator and PrintReads for base quality score recalibration on those initial sites for GLM SNP and then recalled variants with Unified Genotyper with the parameter “—output_mode EMIT_ALL_SITES.” We merged and sorted all of the calls and then ran Variant Filtration with the parameters “QD < 2.0, FS > 60.0, MQ < 40.0.” Next, we removed any base that had less than a minimum genotype quality of 50, a minimum percent alternate allele (AD) of 80%, or a minimum depth of 10. Finally, we removed any positions that were called by both GLMs (i.e., incompatible indels and SNPs), any marked as “LowQual” by GATK, any nested indels, and any sites that did not include a PASS flag. The final base calls covered >95% of the genome for any given isolate. We then categorized every single base between the serial isolates and annotated those changes using the General Feature Files (GFF).

Variant Call format (VCF) files generated by GATK were compared among isolates from same patient. All the identified SNPs between two adjacent time points in serial isolates were validated by manual inspection of read alignments using igvtools (79). All indels located in the coding regions were also verified manually using igvtools.

### Phylogenetic analysis.

In total, 503,457 positions (2.7% of the genome) were called a reference or an SNP (i.e., nonambiguous) position in every VCF and included a variant in at least one isolate. A FASTA file of these positions was created and converted into phylip format, and a phylogenetic tree was generated using RAxML v7.7.8 (80) with 1,000 bootstrap replications. RAxML was run with the generalized time-reversible (GTR) and category (CAT) rate approximation with final evaluation of the tree using GTR plus gamma-distributed rates.

### Large genomic deletion and duplication region identification.

To discover large deletions in the genomes, we applied multiple deletion detection methods, including DELLY (version 0.0.9) (81), BreakDancer (version 1.1) (82), Pindel (version 0.2.4t) (83), and CNVnator (0.2.7) (84). All candidate duplication regions were identified using CNVnator. The read realignment files generated by GATK were followed by duplicate read removal using Picard tools and then processed using all the aforementioned software according to the following settings. A bin size of 50 bp was used for CNVnator. All the raw calls were filtered for the size (<100 bp), call region around centromeres (± 200 bp), and repetitive sequences (50% overlapping). The average read depth of each copy number variation (CNV) call was calculated and compared with the average read depth of the relevant chromosome. We retained only the deletion regions with <20% read depth and duplication regions with <180% read depth for the chromosome. Default settings were used for DELLY, BreakDancer, and Pindel, and the following filters were set for DELLY and BreakDancer. All raw deletion calls with quality scores of <20 or supporting reads of <5 were removed from DELLY, and deletion calls with quality scores of <90 and supporting reads of <5 were removed from the results of BreakDancer.

All the deletion calls from each of the four software methods were merged, and *de novo* assembly of these regions was performed to verify the deletion calls. Briefly, Illumina reads falling into the intervals [(START_INNER −500 bp, START_INNER +100 bp) and (END_INNER −100 bp, END_INNER +500 bp)] for deletion calls were obtained from the bam files using SAMtools and assembled using Velvet (version 1.2.07) according to the insertion length of sequencing libraries. Next, the generated contigs were aligned against the putative deletion regions of the corresponding genomes with 200-bp flanking sequences using cross match (version 0.990319) (http://www.phrap.org). If pairwise alignment indicated the existence of the deletion and if the length of the aligned contig differed by <10% from the expected length, the deletion call was accepted. Identified deletion regions were compared between the isolates from the same patient, and variant deletions were manually checked using igvtools.

### Experimental investigation of the duplication regions.

Genomic DNA of RSA-MW-1746 and RSA-MW-3615 was extracted as described above. Two primers were designed within 100 bp of the duplication breakpoints. The primer sequences of the RSA-MW-1746 duplication region were 5′-CCATCGTCCGTCGGAATCAGTC (left) and 5′-CTTCGAGAACACCTTGAGGACC (right), and the RSA-MW-3615 primer sequences were 5′-GAAGAAATTGGTGGATGCAAGC (left) and 5′-GCCACTCATCTCATTGTTCATC (right). The TagMap assay was performed as previously described (45). After removal of the adaptor and primer sequences, all the sequence reads within 20 bp of the duplication breakpoints were selected and aligned to the *C. neoformans* var. *grubii* H99 genome. The putative insertion region of the extra copies needed to have both ends perfectly aligned by the reads from the two duplication ends. To identify tandem duplication of the duplicated regions, artificial sequences were created using the 100-bp sequences from both duplication ends and concatenated from the 3′ end sequence in 5′-to-3′ order and from the 5′ end sequences in 5′-to-3′ order. Since the sequence reads could be aligned to the middle of the artificial sequence, formation of the tandem duplication in the duplication region was confirmed (Fig. 3B).

### *Galleria mellonella* infections.

The virulence of the isolates was evaluated by monitoring the survival of infected *Galleria mellonella* larvae (Vanderhorst, Inc., St. Marys, OH), as previously described (85). Healthy larvae weighing between 200 mg and 400 mg were used for each experiment. For each isolate, an inoculum of ~50,000 cells (10 μl [5 × 10^6^ CFU/ml]) was injected into the larvae, and 15 to 20 larvae were used in each test. Infected larvae were placed in petri dishes and incubated at 37°C in the dark. The number of surviving larvae was recorded daily for 10 days postinoculation. Each experiment was repeated three times. The survival data were analyzed using the Kaplan-Meier method with GraphPad Prism software.

### Analyses of phenotypes.

The recovered freezer stock of each isolate was streak-plated on YPD agar and incubated at 37°C for 36 to 48 h. A single colony was selected and transferred to three wells across two 96-well plates containing 200 to 300 µl of YPD broth (BD Difco) and incubated for 24 to 48 h at 30C. The colonies were then pin replicated into 384-well plates containing 80 µl of YPD broth (BD Difco) and incubated for 24 to 48 h at 30°C. The liquid culture was then transferred to rectangular agar plates (Greiner Bio-One, catalog no. 781186) at a density of 1,536 colonies per plate using a 384-pin replicating tool with a BM5 robot (S&P Robotics Inc., Ontario, Canada). To minimize any effect of the location of the colonies on the plate (e.g., edge versus center or proximity of colonies), each colony from the 384-well microplate was replicated 24 times (on separate plates [6 sets of 4 blocks]) (see Fig. S2A in the supplemental material). Moreover, for each test, we used two technical replicates, at least three independent tests were performed for each set of conditions (Fig. S2B), and each batch experiment included a control testing of growth at 30°C on YPD. Images of each plate were collected using the same conditions such as aperture and exposure time once a day over 3 days.

The images of colony growth for all testing plates were analyzed using SGATools (49, 86) (Fig. S2C) (see below). Except for the production of melanin by the isolates on l-3,4-dihydroxyphenylalanine (l-DOPA) plates, which was measured by colony color, the growth of each isolate under various conditions was quantified by colony size (see Table S6 in the supplemental material). To minimize system error caused by variations in colony size or color, we calculated averages of the values within the 25% to 75% range for 24 replicates, and then Pearson product-moment correlation coefficients (known as *r*) between two technical replicates within all the middle quadrants were calculated. Tests with an *r* score of less than 0.95 were removed from further analysis, and each set of conditions was required to have three valid independent tests (Fig. S3 and S4).

10.1128/mBio.00166-17.3 FIG S3  Reproducibility of serial isolates cultured on YPD plates at 30°C, 37°C, and 39°C. Two replicates per isolate were tested at one time, and three independent tests were performed for each set of conditions. Each dot represents the average value for one isolate on one plate. Coefficient analysis showed that the results of two replicates were highly consistent. Download FIG S3, EPS file, 1.5 MB. Copyright © 2017 Chen et al. 2017 Chen et al. This content is distributed under the terms of the Creative Commons Attribution 4.0 International license.

10.1128/mBio.00166-17.4 FIG S4  Reproducibility of serial isolates cultured on YPD plates with 0.5 g/liter caffeine, 0.03% SDS, and 100 mg/liter l-DOPA at 30°C. Two replicates per isolate were tested at one time, and three independent tests were performed for each set of conditions. Each dot represents the average value for one isolate on one plate. Coefficient analysis showed that the results of two replicates were highly consistent. Download FIG S4, EPS file, 1.5 MB. Copyright © 2017 Chen et al. 2017 Chen et al. This content is distributed under the terms of the Creative Commons Attribution 4.0 International license.

To eliminate the impact of the different growth rates of these isolates, the colony size of each isolate after incubation at 30°C on YPD agar was deemed to represent an isolate’s basal or control rate of growth. For example, the colony sizes of isolates under conditions of incubation at 37°C or 39°C, and in media with SDS or caffeine, were normalized by dividing them by the corresponding colony sizes at 30°C on YPD to obtain a relative growth rate under each stress condition. However, for melanin production, the average colony pigment of the replicates for each isolate was normalized to the colony pigment of the isolate at 30°C on YPD in the same experimental batch. A *t* test was performed to compare the results from incident and relapse isolates from the same patient with the normalized average values from three tests. All the data analyses and statistical tests were performed in R (87).

Cell wall integrity was assessed on 2% agar YPD medium containing 0.5 g/liter caffeine and 0.03% SDS. Melanin was induced by the use of medium composed of 20 g/liter agar, 1 g/liter l-asparagine, 1 g/liter glucose, 3 g/liter KH_2_PO_4_, 0.25 g/liter MgSO_4 ⋅ _7H_2_O, 1 mg/liter thiamine HCl, 5 μg/liter biotin, and 100 mg/liter l-DOPA.

### Data Availability.

The sequencing data of 38 isolates were submitted to SRA under umbrella project (PRJNA371609), and the project accession numbers are PRJNA227958 (RSA-MW-36), PRJNA227967 (RSA-MW-3335), PRJNA227966 (RSA-MW-1340), PRJNA227944 (RSA-MW-3393), PRJNA227950 (RSA-MW-2799), PRJNA227941 (RSA-MW-5913), PRJNA227936 (RSA-MW-506), PRJNA227937 (RSA-MW-3877), PRJNA227935 (RSA-MW-5465), PRJNA227943 (RSA-MW-1485), PRJNA227946 (RSA-MW-4085), PRJNA227948 (RSA-MW-628), PRJNA227955 (RSA-MW-2914), PRJNA227960 (RSA-MW-2163), PRJNA227959 (RSA-MW-3747), PRJNA227949 (RSA-MW-2015), PRJNA227942 (RSA-MW-3474), PRJNA227951 (RSA-MW-1186), PRJNA227953 (RSA-MW-3179), PRJNA227965 (RSA-MW-913), PRJNA227940 (RSA-MW-2967), PRJNA227957 (RSA-MW-1052), PRJNA227968 (RSA-MW-3156), PRJNA227952 (RSA-MW-1746), PRJNA227956 (RSA-MW-3615), PRJNA227934 (RSA-MW-1281), PRJNA227938 (RSA-MW-2645), PRJNA227969 (RSA-MW-852), PRJNA227954 (RSA-MW-3316), PRJNA227970 (RSA-MW-4119), PRJNA227963 (RSA-MW-2364), PRJNA227945 (RSA-MW-3580), PRJNA227975 (RSA-MW-2399), PRJNA227972 (RSA-MW-4243), PRJNA227973 (RSA-MW-500), PRJNA227976 (RSA-MW-2343), PRJNA227974 (RSA-MW-3980), and PRJNA227971 (RSA-MW-6610). Sequences of the nuclear genome and General Feature Files (GFF) for *C. gattii* isolates VGI WM276 (74), VGIV IND107 (75), and *C. neoformans* var. *grubii* isolate VNI H99 (5) are available at NCBI (GenBank project accession numbers GCA_000185945.1, GCA_000835755.1, and GCA_000149245.3, respectively).
