# Supplementary material for: Microevolution of Serial Clinical Isolates of Cryptococcus neoformans var. grubii and C. gattii
Source: mBio. 2017 Mar 7;8(2):e00166-17. doi: 10.1128/mBio.00166-17 (PMC5340869; doi:10.1128/mBio.00166-17)
Supplement: TABLE S1 [file mbo001173217st1.pdf]

Isolate Information							Patient Information			Treatment
Isolate	Case Number	Infection Type	Species	Molecular type	Isolation Year	Days after Initial isolate	Gender	Age	HIV	Rx
RSA-MW-36<br>RSA-MW-3335	*1*	I (incident)<br>R (relapse)	*Cng*[a]	VNI	2005	238	F	24	+	FLZ[c] + AMP[d]
RSA-MW-1340<br>RSA-MW-3393	8	I<br>R	*Cng*	VNI	2005	144	M	45	+	FLZ
RSA-MW-2799<br>RSA-MW-5913	14	I<br>R	*Cng*	VNI	2006	145	M	27	+	FLZ
RSA-MW-506<br>RSA-MW-3877<br>RSA-MW-5465	15	I<br>R<br>R2	*Cng*	VNI	2006	166<br>223 (57 since R)	F	24	+	FLZ
RSA-MW-1485<br>RSA-MW-4085	22	I<br>R	*Cng*	VNI	2006	151	M	34	+	FLZ + AMP
RSA-MW-628<br>RSA-MW-2914	76	I<br>R	*Cng*	VNI	2008	150	M	39	+	FLZ
RSA-MW-2163<br>RSA-MW-3747	82	I<br>R	*Cng*	VNI	2008	182	F	29	+	AMP
RSA-MW-2015<br>RSA-MW-3474	87	I<br>R	*Cng*	VNI	2008	136	F	29	+	AMP
RSA-MW-1186<br>RSA-MW-3179	7	I<br>R	*Cng*	VNB	2005	137	M	38	Uknown	FLZ
RSA-MW-913<br>RSA-MW-2967	81	I<br>R	*Cng*	VNB	2008	146	M	49	+	FLZ
RSA-MW-1052<br>RSA-MW-3156	5	I<br>R	*Cng*	VNII	2005	153	M	11	+	AMP
RSA-MW-1746<br>RSA-MW-3615	9	I<br>R	*Cng*	VNII	2005	138	F	10	+	FLZ
RSA-MW-1281<br>RSA-MW-2645	45	I<br>R	*Cng*	VNII	2007	131	M	36	Uknown	Uknown
RSA-MW-852		I								

RSA-MW-3316<br>RSA-MW-4119	77	R<br>R2	*Cng*	VNII	2008	182<br>290 (108 since R)	F	40	+	FLZ + AMP
RSA-MW-2364<br>RSA-MW-3580	90	I<br>R	*Cng*	VNII	2008	147	F	40	Uknown	Uknown
RSA-MW-2399<br>RSA-MW-4243	91	I<br>R	*Cg* [b]	VGI	2008	224	M	36	+	AMP
RSA-MW-500<br>RSA-MW-2343	31	I<br>R	*Cg*	VGIV	2007	124	M	34	+	AMP
RSA-MW-3980<br>RSA-MW-6610	67	I<br>R	*Cg*	VGIV	2007	135	M	46	+	AMP

[a] *Cryptococcus neoformans var. grubii*

[b] *Cryptococcus gattii*

[c] Fluconazole

[d] Amphotericin
