# Supplementary figures and images for: Microevolution of Serial Clinical Isolates of Cryptococcus neoformans var. grubii and C. gattii
Source: mBio. 2017 Mar 7;8(2):e00166-17. doi: 10.1128/mBio.00166-17 (PMC5340869; doi:10.1128/mBio.00166-17)

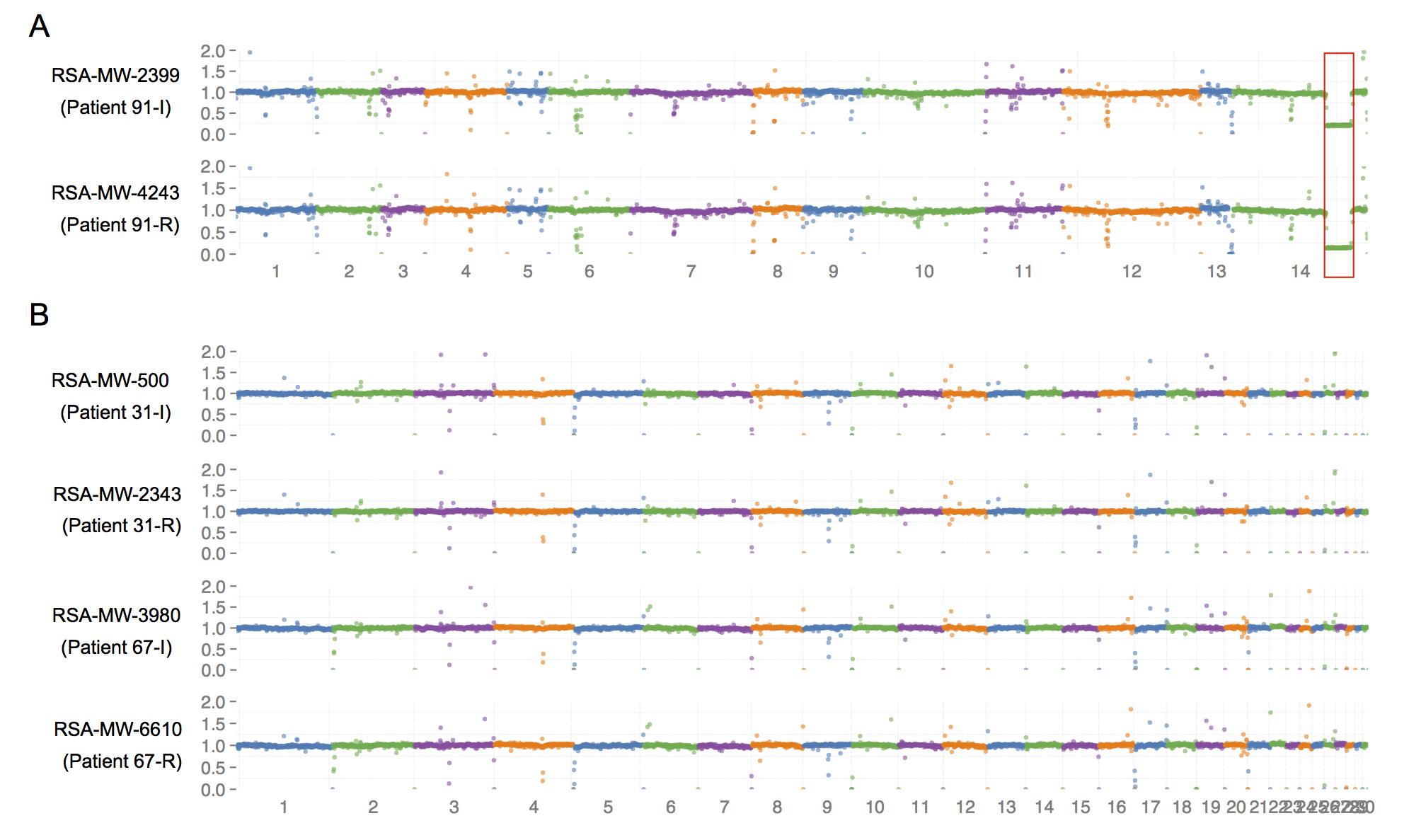

Supplement: FIG S1 [file mbo001173217sf1.tif]
