# Supplementary material for: Microevolution of Serial Clinical Isolates of Cryptococcus neoformans var. grubii and C. gattii
Source: mBio. 2017 Mar 7;8(2):e00166-17. doi: 10.1128/mBio.00166-17 (PMC5340869; doi:10.1128/mBio.00166-17)
Supplement: TABLE S2 [file mbo001173217st2.pdf]

Isolate	Case number	Infection type	Molecular type	Nucleotides aligned	Alignment depth (X)	Reference	SNPs	Breadth of coverage (%)	NCBI BioProject	SRA Accessions
RSA-MW-36	1	I (incident)	VNI	5,422,910,772	295	18,450,491	52,541	98.03	PRJNA227958	SRX400016, SRX400017, SRX400018
RSA-MW-3335		R (relapse)		4,758,717,153	259	18,350,204	52,736	97.50	PRJNA227967	SRX399998, SRX399999, SRX400000
RSA-MW-1340	8	I	VNI	5,175,988,409	282	18,411,641	54,488	97.84	PRJNA227966	SRX399959, SRX399960, SRX399961
RSA-MW-3393		R		4,470,267,773	243	18,396,219	54,372	97.75	PRJNA227944	SRX400001, SRX400002, SRX400003
RSA-MW-2799	14	I	VNI	5,432,310,345	296	18,539,245	11,654	98.29	PRJNA227950	SRX399980, SRX399981, SRX399982
RSA-MW-5913		R		5,690,670,078	310	18,539,360	11,693	98.29	PRJNA227941	SRX400040, SRX400041, SRX400042
RSA-MW-506	15	I	VNI	4,967,765,848	271	18,424,741	51,139	97.89	PRJNA227936	SRX400034, SRX400035, SRX400036
RSA-MW-3877		R		4,988,198,903	272	18,419,855	51,155	97.86	PRJNA227937	SRX400022, SRX400023, SRX400024
RSA-MW-5465		R2		5,437,689,397	296	18,417,762	51,186	97.85	PRJNA227935	SRX400037, SRX400038, SRX400039
RSA-MW-1485	22	I	VNI	4,740,946,087	258	18,528,128	14,783	98.24	PRJNA227943	SRX399962, SRX399963, SRX399964
RSA-MW-4085		R		5,043,788,563	275	18,526,862	14,820	98.24	PRJNA227946	SRX400028, SRX400029, SRX400030
RSA-MW-628	76	I	VNI	4,797,747,409	261	18,415,832	39,118	97.78	PRJNA227948	SRX400043, SRX400044, SRX400045
RSA-MW-2914		R		5,094,256,189	277	18,429,929	39,233	97.85	PRJNA227955	SRX399983, SRX399984, SRX399985
RSA-MW-2163	82	I	VNI	4,937,639,484	269	18,410,076	39,078	97.75	PRJNA227960	SRX399971, SRX399972, SRX399973
RSA-MW-3747		R		4,235,388,612	231	18,403,239	39,006	97.71	PRJNA227959	SRX400019, SRX400020, SRX400021
RSA-MW-2015	87	I	VNI	4,722,550,830	257	18,370,405	54,768	97.62	PRJNA227949	SRX399968, SRX399969, SRX399970
RSA-MW-3474		R		5,168,626,635	282	18,374,009	54,959	97.64	PRJNA227942	SRX400007, SRX400008, SRX400009
RSA-MW-1186	7	I	VNB	4,491,765,623	245	18,092,074	235,352	97.10	PRJNA227951	SRX399950, SRX399951, SRX399952
RSA-MW-3179		R		5,181,752,991	282	18,125,982	235,797	97.28	PRJNA227953	SRX399992, SRX399993, SRX399994
RSA-MW-913	81	I	VNB	5,123,244,324	279	18,117,980	236,645	97.25	PRJNA227965	SRX400049, SRX400050, SRX400051
RSA-MW-2967		R		4,611,224,643	251	18,090,896	236,434	97.10	PRJNA227940	SRX399986, SRX399987, SRX399988
RSA-MW-1052	5	I	VNII	4,348,532,294	237	17,961,197	308,964	96.80	PRJNA227957	SRX399947, SRX399948, SRX399949
RSA-MW-3156		R		5,562,123,581	303	17,928,688	309,713	96.63	PRJNA227968	SRX399989, SRX399990, SRX399991
RSA-MW-1746	9	I	VNII	4,905,163,227	267	17,910,897	309,346	96.53	PRJNA227952	SRX399965, SRX399966, SRX399967
RSA-MW-3615		R		5,079,808,018	277	17,908,192	308,890	96.52	PRJNA227956	SRX400013, SRX400014, SRX400015
RSA-MW-1281	45	I	VNII	4,858,973,140	265	17,923,044	309,275	96.60	PRJNA227934	SRX399953, SRX399954, SRX399955
RSA-MW-2645		R		4,722,605,905	257	17,881,974	309,478	96.38	PRJNA227938	SRX399977, SRX399979, SRX399978

RSA-MW-852	77	I	VNII	5,217,900,956	284	17,845,728	299,879	96.14	PRJNA227969	SRX400046, SRX400047, SRX400048
RSA-MW-3316		R		4,082,792,428	222	17,825,704	299,410	96.03	PRJNA227954	SRX399995, SRX399996, SRX399997
RSA-MW-4119		R2		4,515,558,291	246	17,812,253	299,570	95.96	PRJNA227970	SRX400031, SRX400032, SRX400033
RSA-MW-2364	90	I	VNII	4,348,495,577	237	17,894,615	310,140	96.45	PRJNA227963	SRX399974, SRX399975, SRX399976
RSA-MW-3580		R		4,220,206,100	230	18,036,870	309,092	97.20	PRJNA227945	SRX400010, SRX400011, SRX400012
RSA-MW-2399	91	I	VGI	5,261,555,004	287	17,539,532	93,583	96.05	PRJNA227975	SRX399921, SRX399922, SRX399923
RSA-MW-4243		R		4,795,769,163	261	17,506,719	93,147	95.87	PRJNA227972	SRX399927, SRX399928, SRX399929
RSA-MW-500	31	I	VGIV	5,020,480,326	286	17,475,187	39,749	99.72	PRJNA227973	SRX399930, SRX399931, SRX399932
RSA-MW-2343		R		5,360,211,299	305	17,476,403	39,755	99.73	PRJNA227976	SRX399918, SRX399919, SRX399920
RSA-MW-3980	67	I	VGIV	4,798,123,069	273	17,434,470	52,195	99.56	PRJNA227974	SRX399924, SRX399925, SRX399926
RSA-MW-6610		R		4,177,222,034	238	17,430,511	52,112	99.54	PRJNA227971	SRX399933, SRX399934, SRX399935
