# Supplementary material for: Microevolution of Serial Clinical Isolates of Cryptococcus neoformans var. grubii and C. gattii
Source: mBio. 2017 Mar 7;8(2):e00166-17. doi: 10.1128/mBio.00166-17 (PMC5340869; doi:10.1128/mBio.00166-17)
Supplement: TABLE S3 [file mbo001173217st3.pdf]

	POS	REF (H99)	ALT	case 1	case 14	case 15	case 15R	case 22	case 45	case 5	case 7	case 76	case 77	case 77R	case 8	case 81	case 82	case 87	case 9	case 90
chr1	429251	G	A	-	-	-	-	-	-	-	-	-	-	-	-	-	-	-	-	1-0
chr1	487608	T	C	-	-	-	-	-	-	-	-	-	0-1	1-0	-	-	-	-	-	-
chr1	534510	G	A	-	-	-	-	-	0-1	-	-	-	-	-	-	-	-	-	-	-
chr1	1059033	C	T	-	-	-	-	-	-	-	-	-	0-1	1-0	-	-	-	-	-	-
chr1	1231134	C	T	0-1*	-	-	-	-	-	-	-	-	-	-	-	-	-	-	-	-
chr1	1231135	T	G	0-1	-	-	-	-	-	-	-	-	-	-	-	-	-	-	-	-
chr1	1231136	C	A	0-1	-	-	-	-	-	-	-	-	-	-	-	-	-	-	-	-
chr1	1231137	A	G	0-1	-	-	-	-	-	-	-	-	-	-	-	-	-	-	-	-
chr1	1470454	T	G	-	-	-	-	-	-	1-0	-	-	-	-	-	-	-	-	-	-
chr1	2178025	T	G	-	-	-	-	-	-	-	-	-	1-0	-	-	-	-	-	-	-
chr2	364338	C	T	-	-	-	-	-	-	-	-	-	1-0	-	-	-	-	-	-	-
chr2	636060	C	A	-	-	-	-	-	-	-	-	-	-	-	-	-	-	0-1	-	-
chr2	1108498	A	C	-	-	-	-	-	-	-	1-0	-	-	-	-	-	-	-	-	-
chr3	211642	C	T	-	-	-	-	-	-	-	-	-	-	-	-	-	-	0-1	-	-
chr3	1195416	G	A	-	-	-	-	-	-	-	-	-	-	-	-	1-0	-	-	-	-
chr4	557116	G	T	-	-	-	-	0-1	-	-	-	-	-	-	-	-	-	-	-	-
chr5	165484	G	A	-	-	-	-	-	-	-	-	-	-	-	-	-	-	-	0-1	-
chr5	500970	C	T	-	-	-	-	-	-	-	-	-	-	-	-	0-1	-	-	-	-
chr5	518735	T	C	-	-	-	-	-	-	-	-	-	-	-	-	-	-	-	-	1-0
chr5	1466913	C	T	-	-	-	-	-	-	-	-	-	-	-	-	0-1	-	-	-	-
chr5	1734876	C	T	-	-	-	-	-	-	-	-	-	-	1-0	-	-	-	-	-	-
chr6	1216333	C	T	1-0	-	-	-	-	-	-	-	-	-	-	-	-	-	-	-	-
chr7	95260	C	T	-	-	0-1	1-0	-	-	-	-	-	-	-	-	-	-	-	-	-
chr7	218041	C	T	-	-	-	-	-	-	-	-	-	-	-	-	-	0-1	-	-	-
chr7	345631	T	C	-	0-1	-	-	-	-	-	-	-	-	-	-	-	-	-	-	-
chr7	346774	G	A	-	-	-	-	-	-	-	-	-	-	-	-	-	-	-	-	0-1
chr7	596976	A	T	-	-	-	-	-	-	-	-	-	0-1	1-0	-	-	-	-	-	-
chr7	636692	G	C	-	-	-	-	-	-	-	-	-	-	-	-	0-1	-	-	-	-
chr8	691086	T	A	-	-	-	-	-	-	-	-	-	1-0	-	-	-	-	-	-	-

**chr8**	710291	T	C	-	-	-	-	-	1-0	-	-	-	-	-	-	-	-	-	-	-
**chr8**	1089394	C	T	-	-	-	-	-	-	-	-	-	-	-	-	-	-	0-1	-	-
**chr9**	56975	G	A	-	-	-	-	-	1-0	-	-	-	-	-	-	-	-	-	-	-
**chr9**	442984	G	T	-	-	-	-	-	-	-	0-1	-	-	-	-	-	-	-	-	-
**chr10**	275967	G	A	-	-	-	-	-	-	-	-	-	-	-	0-1	-	-	-	-	-
**chr10**	900410	T	C	-	-	1-0	-	-	-	-	-	-	-	-	-	-	-	-	-	-
**chr10**	942308	T	A	-	-	-	-	-	-	-	-	-	-	-	-	-	-	0-1	-	-
**chr11**	682081	G	A	-	-	-	-	-	-	-	-	-	-	-	-	-	-	-	-	0-1
**chr12**	400791	G	C	-	-	-	-	-	-	-	-	-	-	-	-	-	-	-	1-0	-
**chr12**	681560	C	T	-	-	-	-	-	-	-	-	-	-	-	-	0-1	-	-	-	-
**chr12**	763790	C	T	0-1	-	-	-	-	-	-	-	-	-	-	-	-	-	-	-	-
**chr12**	591802	T	A	-	-	-	1-0	-	-	-	-	-	-	-	-	-	-	-	-	-
**chr14**	102737	G	T	-	-	-	-	-	0-1	-	-	-	-	-	-	-	-	-	-	-
**chr14**	609467	A	G	-	-	-	-	-	-	-	-	-	-	-	-	-	-	-	-	0-1

* The two numbers stand for the single nucleotide viariations (SNV) of the two samples from adjacent time points (initial -> relapse or relapse 1 -> relapse 2) of same patient. "0" stands for the SNV is the same as reference genome (REF), "1" stands for the SNV is the same as the alternative (ALT).

	POS	REF (H99)	ALT	Annotation	Protein change
chr1	429251	G	A	CDS,CNAG_00156, Crz1/Sp1	Gln -> Stop
chr1	487608	T	C	intergenic -- prev_gene]: CNAG_00178 DNA repair protein REV1 (-) -- next_gene: CNAG_00179 guanine nucleotide-binding protein subunit alpha (+)	
chr1	534510	G	A	CDS,CNAG_07316,hydroxyacid-oxoacid transhydrogenase	Pro -> Leu
chr1	1059033	C	T	intergenic -- prev_gene: CNAG_00402 translation initiation factor 4G (-) -- next_gene: CNAG_00403 mitochondrial protein (+)	
chr1	1231134	C	T	CDS,CNAG_07362,nucleolin	Glu -> Ser
chr1	1231135	T	G	CDS,CNAG_07362,nucleolin	Glu -> Ser
chr1	1231136	C	A	CDS,CNAG_07362,nucleolin	Glu -> Ser
chr1	1231137	A	G	CDS,CNAG_07362,nucleolin	
chr1	1470454	T	G	intron,CNAG_00570, PKR1	
chr1	2178025	T	G	CDS,CNAG_00822,ribosome assembly protein SQT1	Phe -> Ser
chr2	364338	C	T	CDS,CNAG_03622	
chr2	636060	C	A	intergenic -- prev_gene: CNAG_03726 hypothetical protein (+) -- next_gene: CNAG_03727 hypothetical protein (+)	
chr2	1108498	A	C	CDS,CNAG_03890,protein lysine methyltransferase SET5	Leu -> Arg
chr3	211642	C	T	CDS,CNAG_03013,OPT family small oligopeptide transporter	Arg -> Stop
chr3	1195416	G	A	CDS,CNAG_07108,tip120-family protein	Ala -> Ser
chr4	557116	G	T	intron,CNAG_05148,beta-1,2-xylosyltransferase 1	
chr5	165484	G	A	CDS,CNAG_06819,DNA helicase INO80	Leu -> Phe
chr5	500970	C	T	intergenic -- prev_gene: CNAG_01361 importin alpha subunit (+) -- next_gene: CNAG_01360 pumilio domain-containing protein c (-)	
chr5	518735	T	C	CDS,CNAG_01354,hypothetical protein	
chr5	1466913	C	T	intergenic -- prev_gene: CNAG_01001 hypothetical protein (+) -- next_gene: CNAG_01000 nuclear pore complex protein Nup155 (+)	
chr5	1734876	C	T	CDS,CNAG_00883,transcription factor	Gln-> Stop
chr6	1216333	C	T	intron,CNAG_02093,hypothetical protein	
chr7	95260	C	T	CDS,CNAG_06552,CAMK/CAMKL/AMPK protein kinase	

chr7	218041	C	T	CDS,CNAG_06592,ubiquitin-conjugating enzyme E2 G1	His -> Tyr
chr7	345631	T	C	intron,CNAG_06637,ubiquitin carboxyl-terminal hydrolase 22/27/51	
chr7	346774	G	A	CDS,CNAG_06637,ubiquitin carboxyl-terminal hydrolase 22/27/51	Gln -> Stop
chr7	596976	A	T	p3UTR,CNAG_07667,HAL protein kinase,-	
chr7	636692	G	C	CDS,CNAG_05695,glucosamine 6-phosphate N-acetyltransferase	Glu -> Gln
chr8	691086	T	A	CDS,CNAG_03341,minichromosome maintenance protein 2	Lle -> Leu
chr8	710291	T	C	intergenic -- prev_gene: CNAG_03346 hypothetical protein (-) -- next_gene: CNAG_03347 ATP-dependent Clp protease ATP-binding subunit ClpB (-)	
chr8	1089394	C	T	CDS,CNAG_03486,peptidyl-prolyl cis-trans isomerase B	Gly -> Ser
chr9	56975	G	A	CDS,CNAG_04118,CMGC/CDK/CRK7 protein kinase	Pro -> Leu
chr9	442984	G	T	p5UTR,CNAG_04270,syntaxin 8,-	
chr10	275967	G	A	intergenic -- prev_gene: CNAG_04837 hypothetical protein (-) -- next_gene: CNAG_04836 nuclear protein (+)	
chr10	900410	T	C	intergenic -- prev_gene: CNAG_04609 argonaute (+) -- next_gene: CNAG_04608 signal peptidase I (+)	
chr10	942308	T	A	CDS,CNAG_04591,hypothetical protein	Met -> Leu
chr11	682081	G	A	CDS,CNAG_01711,glycerol-3-phosphate O-acyltransferase/dihydroxyacetone phosphate acyltransferase	Ala -> Val
chr12	400791	G	C	CDS,CNAG_06136,hypothetical protein	
chr12	681560	C	T	p3UTR,CNAG_06227,fanconi-associated nuclease 1	
chr12	763790	C	T	CDS,CNAG_07011,hypothetical protein	
chr12	591802	T	A	intron,CNAG_06197,hypothetical protein	
chr14	102737	G	T	CDS,CNAG_05366,translation initiation factor 2A	Ala -> Ser
chr14	609467	A	G	CDS,CNAG_05552,transcription activator snf2l1	Leu -> Pro
