# Supplementary material for: Microevolution of Serial Clinical Isolates of Cryptococcus neoformans var. grubii and C. gattii
Source: mBio. 2017 Mar 7;8(2):e00166-17. doi: 10.1128/mBio.00166-17 (PMC5340869; doi:10.1128/mBio.00166-17)
Supplement: TABLE S4 [file mbo001173217st4.pdf]

	POS	REF (R265)	ALT	case 31	case 67	Annotation	Protein change
**supercont2.5**	176915	T	C	0-1	-	CDS,I308_02136,TCO1	Met -> Val
**supercont2.5**	177510	T	A	1-0	-	CDS,I308_02136,TCO1	Lys -> Val
**supercont2.7**	706023	G	A	0-1	-	CDS,I308_03093,tRNA-dihydrouridine synthase 1	
**supercont2.14**	85503	T	G	-	0-1	intergenic -- prev_gene(81757-84337)[<-(1166)]: I308_04861 inositol/phosphatidylinositol phosphatase (+) -- next_gene(85769-88804)[(266)-->]: I308_04862 glucose oxidase (+)	
**supercont2.27**	61569	A	G	0-1	-	intron,I308_06655,D-3-phosphoglycerate dehydrogenase	
