# Supplementary material for: Microevolution of Serial Clinical Isolates of Cryptococcus neoformans var. grubii and C. gattii
Source: mBio. 2017 Mar 7;8(2):e00166-17. doi: 10.1128/mBio.00166-17 (PMC5340869; doi:10.1128/mBio.00166-17)
Supplement: TABLE S6 [file mbo001173217st6.pdf]

Isolate	MIC, FLZ (µg/ml)	37ºC YPD				39ºC YPD				YPD + 0.03% SDS			
		Rep1[a]	Rep2	Rep3	Mean	Rep1	Rep2	Rep3	Mean	Rep1	Rep2	Rep3	Mean
RSA-MW-36	4	1.801	1.812	1.482	1.698	1.717	1.825	1.400	1.647	2.023	2.750	1.738	2.170
RSA-MW-3335	4	1.663	1.864	1.532	1.687	0.972	1.112	1.124	1.069	1.935	2.543	1.863	2.114
RSA-MW-1340	4	0.811	1.029	0.403	0.748	0.000	0.000	0.000	0.000	1.146	0.708	0.161	0.671
RSA-MW-3393	8	1.892	2.161	1.481	1.845	1.068	1.011	0.966	1.015	1.587	2.229	1.262	1.693
RSA-MW-2799	4	1.166	1.310	0.650	1.042	0.043	0.593	0.089	0.242	1.596	2.572	1.418	1.862
RSA-MW-5913	8	1.394	1.519	0.883	1.265	0.928	1.129	0.463	0.840	1.700	2.735	1.524	1.986
RSA-MW-506	2	1.777	2.247	1.183	1.736	1.591	2.366	1.259	1.738	0.000	0.000	0.000	0.000
RSA-MW-3877	8	2.051	1.907	1.613	1.857	1.860	1.870	1.374	1.702	1.809	1.871	1.426	1.702
RSA-MW-1485	8	1.862	2.049	1.335	1.749	1.517	2.181	1.320	1.673	1.136	2.779	1.720	1.878
RSA-MW-4085	16	1.566	2.025	1.111	1.567	0.006	0.063	0.059	0.043	0.637	2.007	0.532	1.058
RSA-MW-628	4	1.962	2.056	1.655	1.891	1.941	2.385	1.661	1.996	1.780	2.335	1.590	1.902
RSA-MW-2914	8	1.895	1.924	1.445	1.755	1.601	1.487	1.181	1.423	1.815	2.338	1.304	1.819
RSA-MW-2163	4	2.256	2.104	1.522	1.961	2.372	2.621	1.805	2.266	2.079	2.871	1.777	2.242
RSA-MW-3747	8	2.136	2.080	1.789	2.002	2.301	2.403	1.964	2.223	2.274	2.726	2.181	2.393
RSA-MW-2015	4	1.944	1.781	1.281	1.669	1.866	2.169	1.445	1.827	1.706	2.300	1.552	1.853
RSA-MW-3474	4	2.005	1.797	1.433	1.745	2.205	1.901	1.416	1.841	2.176	2.291	1.440	1.969
RSA-MW-1052	0.25	1.312	1.637	1.699	1.549	0.000	0.000	0.000	0.000	2.741	3.083	4.037	3.287
RSA-MW-3156	0.5	1.842	1.675	1.560	1.692	0.000	0.001	0.123	0.041	2.998	2.283	1.119	2.133
RSA-MW-1746	8	1.674	1.833	1.337	1.614	0.717	0.726	0.371	0.604	1.942	2.172	1.542	1.885
RSA-MW-3615	128	1.440	1.432	1.123	1.332	0.000	0.000	0.000	0.000	1.925	1.969	1.616	1.837
RSA-MW-1281	1	1.690	2.030	0.824	1.515	0.003	0.387	0.032	0.141	3.091	5.174	1.973	3.413
RSA-MW-2645	8	1.754	1.612	1.262	1.543	0.017	1.038	0.501	0.518	3.319	3.771	2.884	3.324
RSA-MW-852	2	1.978	1.516	1.112	1.535	0.664	0.647	0.066	0.459	0.000	0.000	0.000	0.000
RSA-MW-3316	4	1.900	1.696	1.549	1.715	1.953	1.838	1.670	1.820	2.324	2.346	1.920	2.197
RSA-MW-2364	2	1.446	1.875	0.999	1.440	0.098	1.037	0.383	0.506	0.000	0.000	0.000	0.000
RSA-MW-3580	2	1.613	1.906	1.210	1.576	0.303	1.453	0.524	0.760	2.915	3.723	2.161	2.933
RSA-MW-1186	1	1.932	1.736	1.549	1.739	2.094	1.963	1.687	1.915	1.792	2.086	1.552	1.810
RSA-MW-3179	16	2.003	1.820	1.754	1.859	2.285	1.425	1.950	1.887	2.089	2.226	1.893	2.069

RSA-MW-913	4	1.710	1.881	1.640	1.744	1.629	1.875	1.633	1.713	2.024	2.884	2.143	2.350
RSA-MW-2967	64	1.880	2.435	1.180	1.832	0.805	0.616	0.356	0.592	1.925	4.431	1.571	2.642
RSA-MW-2399	4	2.238	1.948	1.904	2.030	1.458	1.391	1.453	1.434	0.465	0.705	0.114	0.428
RSA-MW-4243	8	1.892	1.575	1.327	1.598	1.187	1.131	0.839	1.052	0.000	0.000	0.000	0.000
RSA-MW-3980	4	1.385	1.120	1.028	1.178	0.000	0.049	0.000	0.016	2.785	2.571	1.909	2.422
RSA-MW-6610	4	1.515	1.330	1.127	1.324	0.001	0.010	0.002	0.004	2.625	2.014	2.122	2.254
RSA-MW-500	2	1.479	1.200	0.972	1.217	0.330	0.214	0.142	0.229	0.000	0.000	0.000	0.000
RSA-MW-2343	1	0.992	0.688	0.548	0.743	0.002	0.001	0.001	0.001	0.000	0.000	0.000	0.000

[a] Average relative colony sizes compared to controls (see Materials and Methods)

Serial isolates with 4X MIC resistence changes between incident and relapse isolates

Serial isolates with significant ($p<0.05$) phenotypic changes between incident and relapse isolates

Serial isolates with extreme significant ($p<0.01$) phenotypic changes between incident and relapse isolates

Isolate	YPD + 0.5 g/L Caffine				YPD + 100mg/L L-DOPA			
	Rep1	Rep2	Rep3	Mean	Rep1	Rep2	Rep3	Mean
RSA-MW-36	1.157	1.118	0.930	1.068	0.407	0.255	0.412	0.358
RSA-MW-3335	1.415	1.214	1.200	1.276	0.523	0.440	0.514	0.492
RSA-MW-1340	1.002	1.880	0.901	1.261	0.298	0.251	0.187	0.245
RSA-MW-3393	1.633	1.713	1.259	1.535	0.378	0.370	0.687	0.478
RSA-MW-2799	1.085	1.482	0.814	1.127	0.310	0.261	0.306	0.292
RSA-MW-5913	1.296	1.941	1.253	1.496	0.166	0.252	0.229	0.216
RSA-MW-506	2.031	2.665	1.295	1.997	0.364	0.379	0.349	0.364
RSA-MW-3877	1.547	1.443	1.205	1.398	0.325	0.293	0.259	0.292
RSA-MW-1485	0.928	1.452	0.946	1.108	0.372	0.310	0.265	0.315
RSA-MW-4085	1.632	2.703	1.326	1.887	0.363	0.244	0.383	0.330
RSA-MW-628	1.281	1.361	0.849	1.164	0.308	0.321	0.407	0.345
RSA-MW-2914	1.500	1.808	1.189	1.499	0.300	0.241	0.487	0.343
RSA-MW-2163	0.900	0.824	0.595	0.773	0.317	0.239	0.285	0.281
RSA-MW-3747	0.979	0.945	0.673	0.866	0.249	0.252	0.484	0.328
RSA-MW-2015	1.280	1.208	1.029	1.173	0.210	0.208	0.161	0.193
RSA-MW-3474	1.555	1.111	0.975	1.214	0.112	0.138	0.251	0.167
RSA-MW-1052	0.637	0.036	0.183	0.285	0.618	0.380	0.737	0.578
RSA-MW-3156	0.728	0.078	0.209	0.338	0.351	0.276	0.396	0.341
RSA-MW-1746	1.219	0.812	0.880	0.970	0.169	0.183	0.168	0.173
RSA-MW-3615	1.012	0.747	0.857	0.872	0.155	0.170	0.225	0.184
RSA-MW-1281	1.143	2.212	1.194	1.516	0.503	0.410	0.724	0.546
RSA-MW-2645	1.050	1.058	0.624	0.911	0.304	0.355	0.753	0.471
RSA-MW-852	0.034	0.004	0.004	0.014	0.403	0.521	0.482	0.468
RSA-MW-3316	1.195	1.000	1.011	1.069	0.443	0.365	0.616	0.475
RSA-MW-2364	0.889	1.220	0.879	0.996	0.296	0.229	0.450	0.325
RSA-MW-3580	1.487	1.840	1.212	1.513	0.275	0.257	0.371	0.301
RSA-MW-1186	1.177	1.121	0.933	1.077	0.275	0.221	0.337	0.278
RSA-MW-3179	1.239	1.182	1.052	1.158	0.350	0.275	0.188	0.271
RSA-MW-913	1.188	1.332	1.126	1.216	0.155	0.171	0.131	0.152

RSA-MW-2967	2.357	3.791	2.358	2.836	0.261	0.304	0.388	0.318
RSA-MW-2399	1.502	1.130	1.317	1.316	0.109	0.113	0.132	0.118
RSA-MW-4243	1.719	1.077	1.369	1.388	0.198	0.199	0.166	0.188
RSA-MW-3980	2.678	1.976	1.969	2.208	0.083	0.108	0.067	0.086
RSA-MW-6610	2.412	1.566	2.246	2.075	0.045	0.027	0.075	0.049
RSA-MW-500	2.179	1.333	1.540	1.684	0.158	0.180	0.167	0.168
RSA-MW-2343	2.110	1.753	1.538	1.800	0.181	0.164	0.206	0.184
