# Supplementary material for: Microevolution of Serial Clinical Isolates of Cryptococcus neoformans var. grubii and C. gattii
Source: mBio. 2017 Mar 7;8(2):e00166-17. doi: 10.1128/mBio.00166-17 (PMC5340869; doi:10.1128/mBio.00166-17)
Supplement: TABLE S5 [file mbo001173217st5.pdf]

| Strain | Exp ID | Day 1 | | | Day 2 | | | Day 3 | | | Day 4 | | | Day 5 | | | Day 6 | | | Day 7 | | | Day 8 | | | Day 9 | | | Day 10 | | |
|---|---|---|---|---|---|---|---|---|---|---|---|---|---|---|---|---|---|---|---|---|---|---|---|---|---|---|---|---|---|---|---|
| | | **D** | **A** | **C** | **D** | **A** | **C** | **D** | **A** | **C** | **D** | **A** | **C** | **D** | **A** | **C** | **D** | **A** | **C** | **D** | **A** | **C** | **D** | **A** | **C** | **D** | **A** | **C** | **D** | **A** | **C** |
| **PBS** | **1** | 0 | 15 | 0 | 0 | 15 | 0 | 0 | 15 | 0 | 1 | 14 | 0 | 0 | 14 | 0 | 0 | 14 | 0 | 1 | 11 | 2 | 0 | 7 | 4 | 1 | 6 | 0 | 0 | 4 | 2 |
| | **2** | 0 | 15 | 0 | 0 | 15 | 0 | 1 | 14 | 0 | 0 | 14 | 0 | 0 | 14 | 0 | 0 | 14 | 0 | 0 | 10 | 4 | 0 | 8 | 2 | 0 | 7 | 1 | 0 | 2 | 5 |
| | **3** | 0 | 15 | 0 | 0 | 15 | 0 | 0 | 15 | 0 | 0 | 15 | 0 | 0 | 15 | 0 | 0 | 15 | 0 | 1 | 14 | 0 | 1 | 10 | 3 | 0 | 10 | 0 | 0 | 7 | 3 |
| | **4** | 0 | 15 | 0 | 0 | 15 | 0 | 0 | 15 | 0 | 0 | 15 | 0 | 0 | 14 | 1 | 1 | 13 | 0 | 1 | 11 | 1 | 0 | 9 | 2 | 1 | 6 | 2 | 0 | 5 | 1 |
| | **5** | 0 | 15 | 0 | 0 | 15 | 0 | 0 | 15 | 0 | 0 | 15 | 0 | 0 | 15 | 0 | 0 | 14 | 1 | 0 | 12 | 2 | 0 | 10 | 2 | 0 | 8 | 2 | 1 | 7 | 0 |
| | **6** | 0 | 15 | 0 | 0 | 15 | 0 | 0 | 15 | 0 | 0 | 15 | 0 | 0 | 15 | 0 | 0 | 15 | 0 | 0 | 14 | 1 | 5 | 8 | 1 | 0 | 5 | 3 | 2 | 3 | 0 |
| | **7** | 0 | 15 | 0 | 0 | 15 | 0 | 0 | 15 | 0 | 0 | 15 | 0 | 0 | 5 | 0 | 0 | 14 | 1 | 1 | 11 | 2 | 0 | 8 | 3 | 1 | 6 | 1 | 0 | 5 | 1 |
| | **8** | 0 | 15 | 0 | 1 | 14 | 0 | 0 | 14 | 0 | 0 | 14 | 0 | 2 | 12 | 0 | 1 | 10 | 1 | 1 | 9 | 0 | 1 | 8 | 0 | 1 | 6 | 1 | 0 | 6 | 0 |
| | **9** | 0 | 15 | 0 | 0 | 15 | 0 | 0 | 15 | 0 | 0 | 15 | 0 | 0 | 15 | 0 | 0 | 15 | 0 | 0 | 15 | 0 | 0 | 11 | 4 | 1 | 9 | 1 | 0 | 6 | 3 |
| **H99** | **1** | 0 | 15 | 0 | 0 | 15 | 0 | 0 | 15 | 0 | 11 | 4 | 0 | 4 | 0 | 0 | 0 | 0 | 0 | 0 | 0 | 0 | 0 | 0 | 0 | 0 | 0 | 0 | 0 | 0 | 0 |
| | **2** | 1 | 15 | 0 | 0 | 15 | 0 | 4 | 11 | 0 | 8 | 3 | 0 | 3 | 0 | 0 | 0 | 0 | 0 | 0 | 0 | 0 | 0 | 0 | 0 | 0 | 0 | 0 | 0 | 0 | 0 |
| | **3** | 0 | 15 | 0 | 0 | 15 | 0 | 3 | 12 | 0 | 11 | 1 | 0 | 0 | 1 | 0 | 1 | 0 | 0 | 0 | 0 | 0 | 0 | 0 | 0 | 0 | 0 | 0 | 0 | 0 | 0 |
| | **4** | 0 | 15 | 0 | 0 | 15 | 0 | 1 | 14 | 0 | 4 | 10 | 0 | 9 | 1 | 0 | 0 | 1 | 0 | 1 | 0 | 0 | 0 | 0 | 0 | 0 | 0 | 0 | 0 | 0 | 0 |
| | **5** | 0 | 15 | 0 | 0 | 15 | 0 | 1 | 14 | 0 | 8 | 6 | 0 | 6 | 0 | 0 | 0 | 0 | 0 | 0 | 0 | 0 | 0 | 0 | 0 | 0 | 0 | 0 | 0 | 0 | 0 |
| | **6** | 0 | 15 | 0 | 0 | 15 | 0 | 1 | 14 | 0 | 5 | 9 | 0 | 9 | 0 | 0 | 0 | 0 | 0 | 0 | 0 | 0 | 0 | 0 | 0 | 0 | 0 | 0 | 0 | 0 | 0 |
| | **7** | 0 | 15 | 0 | 0 | 15 | 0 | 0 | 15 | 0 | 9 | 6 | 0 | 4 | 2 | 0 | 2 | 0 | 0 | 0 | 0 | 0 | 0 | 0 | 0 | 0 | 0 | 0 | 0 | 0 | 0 |
| | **8** | 0 | 15 | 0 | 0 | 15 | 0 | 0 | 15 | 0 | 3 | 12 | 0 | 11 | 1 | 0 | 1 | 0 | 0 | 0 | 0 | 0 | 0 | 0 | 0 | 0 | 0 | 0 | 0 | 0 | 0 |
| | **9** | 0 | 15 | 0 | 0 | 15 | 0 | 0 | 15 | 0 | 11 | 4 | 0 | 4 | 0 | 0 | 0 | 0 | 0 | 0 | 0 | 0 | 0 | 0 | 0 | 0 | 0 | 0 | 0 | 0 | 0 |
| **RSA-MW-36** | **2** | 0 | 15 | 0 | 0 | 15 | 0 | 0 | 15 | 0 | 0 | 15 | 0 | 0 | 15 | 0 | 0 | 15 | 0 | 10 | 4 | 1 | 3 | 1 | 0 | 1 | 0 | 0 | 0 | 0 | 0 |
| | **4** | 0 | 15 | 0 | 0 | 15 | 0 | 0 | 15 | 0 | 0 | 15 | 0 | 0 | 15 | 0 | 2 | 13 | 0 | 3 | 10 | 0 | 7 | 3 | 0 | 1 | 2 | 0 | 2 | 0 | 0 |
| | **5** | 0 | 15 | 0 | 0 | 15 | 0 | 0 | 15 | 0 | 0 | 15 | 0 | 0 | 15 | 0 | 0 | 15 | 0 | 2 | 13 | 0 | 5 | 8 | 0 | 6 | 2 | 0 | 2 | 0 | 0 |
| **RSA-MW-3335** | **2** | 0 | 15 | 0 | 0 | 15 | 0 | 3 | 12 | 0 | 10 | 2 | 0 | 2 | 0 | 0 | 0 | 0 | 0 | 0 | 0 | 0 | 0 | 0 | 0 | 0 | 0 | 0 | 0 | 0 | 0 |
| | **4** | 0 | 15 | 0 | 0 | 15 | 0 | 1 | 14 | 0 | 9 | 5 | 0 | 4 | 1 | 0 | 1 | 0 | 0 | 0 | 0 | 0 | 0 | 0 | 0 | 0 | 0 | 0 | 0 | 0 | 0 |
| | **5** | 0 | 15 | 0 | 0 | 15 | 0 | 0 | 15 | 0 | 0 | 15 | 0 | 0 | 15 | 0 | 14 | 1 | 0 | 1 | 0 | 0 | 0 | 0 | 0 | 0 | 0 | 0 | 0 | 0 | 0 |
| **RSA-MW-1052** | **1** | 0 | 15 | 0 | 0 | 15 | 0 | 0 | 15 | 0 | 0 | 15 | 0 | 1 | 14 | 0 | 0 | 14 | 0 | 0 | 14 | 0 | 6 | 8 | 0 | 3 | 5 | 0 | 1 | 4 | 0 |
| | **4** | 0 | 15 | 0 | 0 | 15 | 0 | 1 | 14 | 0 | 0 | 14 | 0 | 0 | 14 | 0 | 0 | 14 | 0 | 1 | 12 | 1 | 6 | 6 | 0 | 1 | 5 | 0 | 4 | 1 | 0 |
| | **5** | 0 | 15 | 0 | 0 | 15 | 0 | 0 | 15 | 0 | 0 | 15 | 0 | 0 | 15 | 0 | 0 | 15 | 0 | 1 | 13 | 1 | 5 | 7 | 1 | 6 | 1 | 0 | 1 | 0 | 0 |
| **RSA-MW-3156** | **2** | 1 | 15 | 0 | 0 | 15 | 0 | 0 | 15 | 0 | 0 | 15 | 0 | 0 | 15 | 0 | 9 | 6 | 0 | 5 | 1 | 0 | 1 | 0 | 0 | 0 | 0 | 0 | 0 | 0 | 0 |
| | **3** | 0 | 15 | 0 | 0 | 15 | 0 | 0 | 15 | 0 | 0 | 15 | 0 | 0 | 15 | 0 | 3 | 12 | 0 | 5 | 7 | 0 | 4 | 3 | 0 | 2 | 1 | 0 | 0 | 1 | 0 |

| | | | | | | | | | | | | | | | | | | | | | | | | | | | | | | | |
|---|---|---|---|---|---|---|---|---|---|---|---|---|---|---|---|---|---|---|---|---|---|---|---|---|---|---|---|---|---|---|---|
| | **5** | 0 | 15 | 0 | 0 | 15 | 0 | 0 | 15 | 0 | 0 | 15 | 0 | 2 | 13 | 0 | 9 | 4 | 0 | 4 | 0 | 0 | 0 | 0 | 0 | 0 | 0 | 0 | 0 | 0 | 0 |
| | **2** | 0 | 15 | 0 | 0 | 15 | 0 | 0 | 15 | 0 | 1 | 14 | 0 | 1 | 14 | 0 | 0 | 13 | 0 | 2 | 9 | 2 | 1 | 7 | 1 | 1 | 5 | 1 | 1 | 3 | 1 |
| **RSA-MW-1186** | **4** | 2 | 13 | 0 | 0 | 13 | 0 | 0 | 13 | 0 | 1 | 12 | 0 | 1 | 11 | 0 | 4 | 7 | 0 | 1 | 5 | 1 | 1 | 3 | 1 | 2 | 1 | 0 | 1 | 0 | 0 |
| | **5** | 0 | 15 | 0 | 0 | 15 | 0 | 0 | 15 | 0 | 0 | 15 | 0 | 0 | 15 | 0 | 4 | 11 | 0 | 3 | 8 | 0 | 2 | 6 | 0 | 4 | 2 | 0 | 0 | 2 | 0 |
| | **1** | 0 | 15 | 0 | 0 | 15 | 0 | 0 | 15 | 0 | 1 | 14 | 0 | 3 | 11 | 0 | 2 | 9 | 0 | 2 | 7 | 0 | 1 | 6 | 0 | 5 | 1 | 0 | 0 | 1 | 0 |
| **RSA-MW-3179** | **4** | 0 | 15 | 0 | 0 | 15 | 0 | 1 | 14 | 0 | 0 | 14 | 0 | 0 | 14 | 0 | 2 | 12 | 0 | 1 | 11 | 0 | 4 | 7 | 0 | 2 | 5 | 0 | 3 | 2 | 0 |
| | **5** | 0 | 15 | 0 | 0 | 15 | 0 | 0 | 15 | 0 | 1 | 14 | 0 | 6 | 8 | 0 | 2 | 6 | 0 | 1 | 5 | 0 | 1 | 4 | 0 | 1 | 3 | 0 | 0 | 3 | 0 |
| | **1** | 0 | 15 | 0 | 0 | 15 | 0 | 5 | 10 | 0 | 8 | 2 | 0 | 2 | 0 | 0 | 0 | 0 | 0 | 0 | 0 | 0 | 0 | 0 | 0 | 0 | 0 | 0 | 0 | 0 | 0 |
| **RSA-MW-1340** | **4** | 0 | 15 | 0 | 0 | 14 | 1 | 0 | 14 | 0 | 4 | 10 | 0 | 8 | 2 | 0 | 2 | 0 | 0 | 0 | 0 | 0 | 0 | 0 | 0 | 0 | 0 | 0 | 0 | 0 | 0 |
| | **6** | 0 | 15 | 0 | 0 | 15 | 0 | 0 | 15 | 0 | 12 | 3 | 0 | 2 | 1 | 0 | 1 | 0 | 0 | 0 | 0 | 0 | 0 | 0 | 0 | 0 | 0 | 0 | 0 | 0 | 0 |
| | **1** | 0 | 15 | 0 | 0 | 15 | 0 | 0 | 15 | 0 | 5 | 10 | 0 | 7 | 3 | 0 | 3 | 0 | 0 | 0 | 0 | 0 | 0 | 0 | 0 | 0 | 0 | 0 | 0 | 0 | 0 |
| **RSA-MW-3393** | **3** | 0 | 15 | 0 | 0 | 15 | 0 | 1 | 14 | 0 | 4 | 10 | 0 | 4 | 6 | 0 | 5 | 1 | 0 | 0 | 1 | 0 | 0 | 1 | 0 | 1 | 0 | 0 | 0 | 0 | 0 |
| | **6** | 0 | 15 | 0 | 0 | 15 | 0 | 0 | 15 | 0 | 6 | 9 | 0 | 8 | 1 | 0 | 1 | 0 | 0 | 0 | 0 | 0 | 0 | 0 | 0 | 0 | 0 | 0 | 0 | 0 | 0 |
| | **2** | 0 | 15 | 0 | 3 | 12 | 0 | 1 | 11 | 0 | 0 | 11 | 0 | 0 | 11 | 0 | 1 | 10 | 0 | 2 | 8 | 0 | 5 | 3 | 0 | 2 | 1 | 0 | 1 | 0 | 0 |
| **RSA-MW-1746** | **4** | 0 | 15 | 0 | 0 | 15 | 0 | 0 | 15 | 0 | 0 | 15 | 0 | 0 | 14 | 1 | 4 | 10 | 0 | 7 | 3 | 0 | 2 | 1 | 0 | 1 | 0 | 0 | 0 | 0 | 0 |
| | **6** | 0 | 15 | 0 | 0 | 15 | 0 | 0 | 15 | 0 | 0 | 15 | 0 | 0 | 15 | 0 | 0 | 15 | 0 | 5 | 10 | 0 | 9 | 1 | 0 | 0 | 1 | 0 | 0 | 1 | 0 |
| | **1** | 2 | 15 | 0 | 0 | 15 | 0 | 0 | 15 | 0 | 0 | 15 | 0 | 0 | 15 | 0 | 1 | 14 | 0 | 5 | 9 | 0 | 5 | 4 | 0 | 4 | 0 | 0 | 0 | 0 | 0 |
| **RSA-MW-3615** | **4** | 0 | 15 | 0 | 0 | 15 | 0 | 0 | 15 | 0 | 0 | 15 | 0 | 0 | 15 | 0 | 0 | 15 | 0 | 1 | 14 | 0 | 7 | 7 | 0 | 4 | 3 | 0 | 3 | 0 | 0 |
| | **6** | 0 | 15 | 0 | 0 | 15 | 0 | 0 | 15 | 0 | 0 | 15 | 0 | 0 | 15 | 0 | 0 | 15 | 0 | 3 | 12 | 0 | 3 | 9 | 0 | 7 | 2 | 0 | 2 | 0 | 0 |
| | **1** | 0 | 15 | 0 | 0 | 15 | 0 | 0 | 15 | 0 | 1 | 14 | 0 | 3 | 11 | 0 | 3 | 8 | 0 | 5 | 3 | 0 | 1 | 2 | 0 | 2 | 0 | 0 | 0 | 0 | 0 |
| **RSA-MW-2799** | **3** | 0 | 15 | 0 | 0 | 15 | 0 | 0 | 15 | 0 | 1 | 14 | 0 | 6 | 8 | 0 | 2 | 6 | 0 | 3 | 3 | 0 | 2 | 0 | 1 | 0 | 0 | 0 | 0 | 0 | 0 |
| | **6** | 0 | 15 | 0 | 0 | 15 | 0 | 0 | 15 | 0 | 1 | 14 | 0 | 0 | 14 | 0 | 1 | 13 | 0 | 9 | 4 | 0 | 4 | 0 | 0 | 0 | 0 | 0 | 0 | 0 | 0 |
| | **2** | 1 | 15 | 0 | 0 | 15 | 0 | 0 | 15 | 0 | 0 | 15 | 0 | 4 | 11 | 0 | 8 | 3 | 0 | 3 | 0 | 0 | 0 | 0 | 0 | 0 | 0 | 0 | 0 | 0 | 0 |
| **RSA-MW-5913** | **4** | 0 | 15 | 0 | 1 | 14 | 0 | 0 | 14 | 0 | 2 | 12 | 0 | 4 | 8 | 0 | 3 | 5 | 0 | 2 | 3 | 0 | 3 | 0 | 0 | 0 | 0 | 0 | 0 | 0 | 0 |
| | **6** | 0 | 15 | 0 | 0 | 15 | 0 | 0 | 15 | 0 | 0 | 15 | 0 | 7 | 8 | 0 | 3 | 5 | 0 | 5 | 0 | 0 | 0 | 0 | 0 | 0 | 0 | 0 | 0 | 0 | 0 |
| | **1** | 0 | 15 | 0 | 0 | 15 | 0 | 0 | 15 | 0 | 0 | 15 | 0 | 0 | 15 | 0 | 0 | 15 | 0 | 1 | 14 | 0 | 0 | 8 | 6 | 0 | 7 | 1 | 1 | 5 | 1 |
| **RSA-MW-506** | **4** | 0 | 15 | 0 | 0 | 15 | 0 | 0 | 15 | 0 | 0 | 15 | 0 | 0 | 15 | 0 | 0 | 13 | 2 | 1 | 12 | 0 | 0 | 10 | 2 | 3 | 7 | 0 | 0 | 6 | 1 |
| | **5** | 0 | 15 | 0 | 0 | 15 | 0 | 0 | 15 | 0 | 0 | 15 | 0 | 0 | 15 | 0 | 0 | 15 | 0 | 1 | 13 | 1 | 0 | 10 | 3 | 0 | 8 | 2 | 0 | 8 | 0 |
| | **2** | 0 | 15 | 0 | 0 | 15 | 0 | 0 | 15 | 0 | 2 | 13 | 0 | 3 | 10 | 0 | 5 | 5 | 0 | 2 | 3 | 0 | 0 | 1 | 2 | 1 | 0 | 0 | 0 | 0 | 0 |
| **RSA-MW-3877** | **3** | 0 | 15 | 0 | 0 | 15 | 0 | 0 | 15 | 0 | 0 | 15 | 0 | 0 | 14 | 1 | 2 | 12 | 0 | 2 | 10 | 0 | 5 | 4 | 1 | 1 | 3 | 0 | 1 | 2 | 0 |

| | | | | | | | | | | | | | | | | | | | | | | | | | | | | | | | |
|---|---|---|---|---|---|---|---|---|---|---|---|---|---|---|---|---|---|---|---|---|---|---|---|---|---|---|---|---|---|---|---|
| | **5** | 0 | 15 | 0 | 0 | 15 | 0 | 0 | 15 | 0 | 0 | 15 | 0 | 0 | 15 | 0 | 0 | 15 | 0 | 3 | 12 | 0 | 1 | 11 | 0 | 3 | 8 | 0 | 3 | 5 | 0 |
| **RSA-MW-1485** | **1** | 0 | 15 | 0 | 0 | 15 | 0 | 0 | 15 | 0 | 4 | 11 | 0 | 2 | 9 | 0 | 8 | 1 | 0 | 1 | 0 | 0 | 0 | 0 | 0 | 0 | 0 | 0 | 0 | 0 | 0 |
| | **4** | 1 | 14 | 0 | 0 | 14 | 0 | 0 | 14 | 0 | 4 | 10 | 0 | 9 | 1 | 0 | 1 | 0 | 0 | 0 | 0 | 0 | 0 | 0 | 0 | 0 | 0 | 0 | 0 | 0 | 0 |
| | **5** | 0 | 15 | 0 | 0 | 15 | 0 | 0 | 15 | 0 | 1 | 14 | 0 | 13 | 1 | 0 | 1 | 0 | 0 | 0 | 0 | 0 | 0 | 0 | 0 | 0 | 0 | 0 | 0 | 0 | 0 |
| **RSA-MW-4085** | **1** | 0 | 15 | 0 | 0 | 15 | 0 | 0 | 15 | 0 | 0 | 15 | 0 | 1 | 14 | 0 | 3 | 11 | 0 | 4 | 7 | 0 | 7 | 0 | 0 | 0 | 0 | 0 | 0 | 0 | 0 |
| | **3** | 0 | 15 | 0 | 0 | 15 | 0 | 0 | 15 | 0 | 0 | 15 | 0 | 0 | 15 | 0 | 2 | 13 | 0 | 2 | 11 | 0 | 8 | 3 | 0 | 2 | 1 | 0 | 1 | 0 | 0 |
| | **5** | 0 | 15 | 0 | 0 | 15 | 0 | 0 | 15 | 0 | 0 | 15 | 0 | 0 | 15 | 0 | 1 | 14 | 0 | 6 | 7 | 1 | 5 | 2 | 0 | 2 | 0 | 0 | 0 | 0 | 0 |
| **RSA-MW-1281** | **2** | 1 | 15 | 0 | 0 | 15 | 0 | 0 | 15 | 0 | 0 | 15 | 0 | 0 | 15 | 0 | 1 | 14 | 0 | 1 | 13 | 0 | 8 | 5 | 0 | 5 | 0 | 0 | 0 | 0 | 0 |
| | **4** | 1 | 14 | 0 | 0 | 14 | 0 | 0 | 14 | 0 | 0 | 14 | 0 | 0 | 14 | 0 | 2 | 12 | 0 | 3 | 9 | 0 | 3 | 6 | 0 | 4 | 2 | 0 | 0 | 2 | 0 |
| | **6** | 0 | 15 | 0 | 0 | 15 | 0 | 0 | 15 | 0 | 0 | 15 | 0 | 0 | 15 | 0 | 0 | 15 | 0 | 0 | 15 | 0 | 6 | 9 | 0 | 7 | 2 | 0 | 1 | 1 | 0 |
| **RSA-MW-2645** | **1** | 0 | 15 | 0 | 0 | 15 | 0 | 0 | 15 | 0 | 0 | 15 | 0 | 0 | 15 | 0 | 6 | 9 | 0 | 5 | 4 | 0 | 2 | 2 | 0 | 0 | 2 | 0 | 0 | 2 | 0 |
| | **3** | 0 | 15 | 0 | 0 | 15 | 0 | 0 | 15 | 0 | 0 | 15 | 0 | 0 | 15 | 0 | 6 | 9 | 0 | 5 | 4 | 0 | 4 | 0 | 0 | 0 | 0 | 0 | 0 | 0 | 0 |
| | **6** | 0 | 15 | 0 | 0 | 15 | 0 | 0 | 15 | 0 | 0 | 15 | 0 | 1 | 14 | 0 | 0 | 14 | 0 | 6 | 8 | 0 | 7 | 1 | 0 | 1 | 0 | 0 | 0 | 0 | 0 |
| **RSA-MW-628** | **1** | 0 | 15 | 0 | 0 | 15 | 0 | 0 | 15 | 0 | 5 | 10 | 0 | 4 | 6 | 0 | 5 | 1 | 0 | 1 | 0 | 0 | 0 | 0 | 0 | 0 | 0 | 0 | 0 | 0 | 0 |
| | **4** | 0 | 15 | 0 | 0 | 15 | 0 | 0 | 15 | 0 | 0 | 15 | 0 | 8 | 7 | 0 | 4 | 3 | 0 | 2 | 1 | 0 | 0 | 1 | 0 | 1 | 0 | 0 | 0 | 0 | 0 |
| | **6** | 0 | 15 | 0 | 0 | 15 | 0 | 0 | 15 | 0 | 2 | 13 | 0 | 7 | 6 | 0 | 4 | 2 | 0 | 2 | 0 | 0 | 0 | 0 | 0 | 0 | 0 | 0 | 0 | 0 | 0 |
| **RSA-MW-2914** | **1** | 0 | 15 | 0 | 0 | 15 | 0 | 6 | 9 | 0 | 6 | 3 | 0 | 3 | 0 | 0 | 0 | 0 | 0 | 0 | 0 | 0 | 0 | 0 | 0 | 0 | 0 | 0 | 0 | 0 | 0 |
| | **3** | 0 | 15 | 0 | 0 | 15 | 0 | 0 | 15 | 0 | 3 | 11 | 1 | 7 | 4 | 0 | 1 | 3 | 0 | 2 | 1 | 0 | 0 | 1 | 0 | 0 | 1 | 0 | 1 | 0 | 0 |
| | **6** | 0 | 15 | 0 | 0 | 15 | 0 | 0 | 15 | 0 | 4 | 11 | 0 | 7 | 4 | 0 | 2 | 2 | 0 | 1 | 1 | 0 | 1 | 0 | 0 | 0 | 0 | 0 | 0 | 0 | 0 |
| **RSA-MW-852** | **2** | 1 | 15 | 0 | 0 | 15 | 0 | 0 | 15 | 0 | 0 | 15 | 0 | 1 | 14 | 0 | 4 | 9 | 1 | 3 | 6 | 0 | 1 | 5 | 0 | 1 | 4 | 0 | 3 | 1 | 0 |
| | **3** | 0 | 15 | 0 | 0 | 15 | 0 | 0 | 15 | 0 | 0 | 15 | 0 | 4 | 9 | 2 | 2 | 7 | 0 | 2 | 4 | 1 | 2 | 2 | 0 | 1 | 1 | 0 | 0 | 1 | 0 |
| | **5** | 0 | 15 | 0 | 0 | 15 | 0 | 0 | 15 | 0 | 0 | 15 | 0 | 0 | 15 | 0 | 1 | 14 | 0 | 3 | 9 | 2 | 2 | 7 | 0 | 3 | 4 | 0 | 3 | 1 | 0 |
| **RSA-MW-3316** | **2** | 0 | 15 | 0 | 0 | 15 | 0 | 0 | 15 | 0 | 0 | 15 | 0 | 0 | 15 | 0 | 2 | 13 | 0 | 6 | 7 | 0 | 4 | 3 | 0 | 2 | 1 | 0 | 1 | 0 | 0 |
| | **3** | 0 | 15 | 0 | 0 | 15 | 0 | 1 | 14 | 0 | 0 | 14 | 0 | 0 | 14 | 0 | 0 | 14 | 0 | 2 | 12 | 0 | 4 | 8 | 0 | 4 | 4 | 0 | 3 | 1 | 0 |
| | **5** | 0 | 15 | 0 | 0 | 15 | 0 | 0 | 15 | 0 | 0 | 15 | 0 | 0 | 15 | 0 | 0 | 15 | 0 | 2 | 11 | 2 | 5 | 6 | 0 | 3 | 3 | 0 | 3 | 0 | 0 |
| **RSA-MW-913** | **1** | 0 | 15 | 0 | 0 | 15 | 0 | 0 | 15 | 0 | 1 | 14 | 0 | 5 | 9 | 0 | 3 | 6 | 0 | 5 | 1 | 0 | 1 | 0 | 0 | 0 | 0 | 0 | 0 | 0 | 0 |
| | **3** | 0 | 15 | 0 | 0 | 15 | 0 | 0 | 15 | 0 | 2 | 13 | 0 | 5 | 8 | 0 | 8 | 0 | 0 | 0 | 0 | 0 | 0 | 0 | 0 | 0 | 0 | 0 | 0 | 0 | 0 |
| | **5** | 0 | 15 | 0 | 0 | 15 | 0 | 0 | 15 | 0 | 1 | 14 | 0 | 4 | 10 | 0 | 6 | 4 | 0 | 4 | 0 | 0 | 0 | 0 | 0 | 0 | 0 | 0 | 0 | 0 | 0 |
| **RSA-MW-2967** | **2** | 0 | 15 | 0 | 0 | 15 | 0 | 0 | 15 | 0 | 1 | 14 | 0 | 4 | 10 | 0 | 5 | 5 | 0 | 3 | 2 | 0 | 1 | 1 | 0 | 1 | 0 | 0 | 0 | 0 | 0 |
| | **4** | 0 | 15 | 0 | 0 | 15 | 0 | 0 | 15 | 0 | 0 | 14 | 0 | 7 | 7 | 0 | 5 | 2 | 0 | 2 | 0 | 0 | 0 | 0 | 0 | 0 | 0 | 0 | 0 | 0 | 0 |

| | | | | | | | | | | | | | | | | | | | | | | | | | | | | | | | |
|---|---|---|---|---|---|---|---|---|---|---|---|---|---|---|---|---|---|---|---|---|---|---|---|---|---|---|---|---|---|---|---|
| | **5** | 0 | 15 | 0 | 0 | 15 | 0 | 0 | 15 | 0 | 0 | 15 | 0 | 2 | 13 | 0 | 0 | 13 | 0 | 2 | 11 | 0 | 3 | 8 | 0 | 4 | 4 | 0 | 4 | 0 | 0 |
| | **1** | 1 | 15 | 0 | 0 | 15 | 0 | 0 | 15 | 0 | 8 | 7 | 0 | 3 | 4 | 0 | 4 | 0 | 0 | 0 | 0 | 0 | 0 | 0 | 0 | 0 | 0 | 0 | 0 | 0 | 0 |
| **RSA-MW-2163** | **3** | 0 | 15 | 0 | 0 | 15 | 0 | 0 | 15 | 0 | 7 | 8 | 0 | 4 | 4 | 0 | 4 | 0 | 0 | 0 | 0 | 0 | 0 | 0 | 0 | 0 | 0 | 0 | 0 | 0 | 0 |
| | **5** | 0 | 15 | 0 | 0 | 15 | 0 | 0 | 15 | 0 | 3 | 12 | 0 | 0 | 0 | 0 | 12 | 0 | 0 | 0 | 0 | 0 | 0 | 0 | 0 | 0 | 0 | 0 | 0 | 0 | 0 |
| | **2** | 1 | 15 | 0 | 0 | 15 | 0 | 0 | 15 | 0 | 6 | 9 | 0 | 8 | 1 | 0 | 1 | 0 | 0 | 0 | 0 | 0 | 0 | 0 | 0 | 0 | 0 | 0 | 0 | 0 | 0 |
| **RSA-MW-3747** | **3** | 0 | 15 | 0 | 0 | 15 | 0 | 1 | 14 | 0 | 7 | 7 | 0 | 7 | 0 | 0 | 0 | 0 | 0 | 0 | 0 | 0 | 0 | 0 | 0 | 0 | 0 | 0 | 0 | 0 | 0 |
| | **5** | 0 | 15 | 0 | 0 | 15 | 0 | 0 | 15 | 0 | 9 | 6 | 0 | 5 | 1 | 0 | 0 | 1 | 0 | 1 | 0 | 0 | 0 | 0 | 0 | 0 | 0 | 0 | 0 | 0 | 0 |
| | **2** | 1 | 15 | 0 | 0 | 15 | 0 | 0 | 15 | 0 | 0 | 15 | 0 | 4 | 11 | 0 | 11 | 0 | 0 | 0 | 0 | 0 | 0 | 0 | 0 | 0 | 0 | 0 | 0 | 0 | 0 |
| **RSA-MW-2015** | **4** | 0 | 15 | 0 | 0 | 15 | 0 | 0 | 15 | 0 | 0 | 15 | 0 | 7 | 8 | 0 | 7 | 1 | 0 | 1 | 0 | 0 | 0 | 0 | 0 | 0 | 0 | 0 | 0 | 0 | 0 |
| | **5** | 0 | 15 | 0 | 0 | 15 | 0 | 0 | 15 | 0 | 0 | 15 | 0 | 9 | 6 | 0 | 4 | 2 | 0 | 2 | 0 | 0 | 0 | 0 | 0 | 0 | 0 | 0 | 0 | 0 | 0 |
| | **1** | 0 | 15 | 0 | 0 | 15 | 0 | 0 | 15 | 0 | 0 | 15 | 0 | 14 | 1 | 0 | 0 | 1 | 0 | 1 | 0 | 0 | 0 | 0 | 0 | 0 | 0 | 0 | 0 | 0 | 0 |
| **RSA-MW-3474** | **3** | 0 | 15 | 0 | 0 | 15 | 0 | 0 | 15 | 0 | 4 | 11 | 0 | 5 | 6 | 0 | 5 | 1 | 0 | 1 | 0 | 0 | 0 | 0 | 0 | 0 | 0 | 0 | 0 | 0 | 0 |
| | **5** | 0 | 15 | 0 | 0 | 15 | 0 | 0 | 15 | 0 | 4 | 11 | 0 | 11 | 0 | 0 | 0 | 0 | 0 | 0 | 0 | 0 | 0 | 0 | 0 | 0 | 0 | 0 | 0 | 0 | 0 |
| | **2** | 1 | 15 | 0 | 0 | 15 | 0 | 0 | 15 | 0 | 0 | 15 | 0 | 0 | 15 | 0 | 7 | 8 | 0 | 5 | 3 | 0 | 2 | 1 | 0 | 1 | 0 | 0 | 0 | 0 | 0 |
| **RSA-MW-2364** | **3** | 0 | 15 | 0 | 1 | 14 | 0 | 0 | 14 | 0 | 0 | 14 | 0 | 1 | 13 | 0 | 2 | 11 | 0 | 6 | 5 | 0 | 2 | 3 | 0 | 2 | 1 | 0 | 0 | 1 | 0 |
| | **6** | 0 | 15 | 0 | 0 | 15 | 0 | 0 | 15 | 0 | 0 | 15 | 0 | 0 | 15 | 0 | 0 | 15 | 0 | 3 | 12 | 0 | 7 | 5 | 0 | 1 | 4 | 0 | 2 | 2 | 0 |
| | **2** | 1 | 15 | 0 | 0 | 15 | 0 | 0 | 15 | 0 | 0 | 15 | 0 | 0 | 15 | 0 | 6 | 9 | 0 | 7 | 1 | 1 | 1 | 0 | 0 | 0 | 0 | 0 | 0 | 0 | 0 |
| **RSA-MW-3580** | **3** | 0 | 15 | 0 | 0 | 15 | 0 | 0 | 15 | 0 | 0 | 15 | 0 | 0 | 15 | 0 | 3 | 12 | 0 | 5 | 7 | 0 | 5 | 2 | 0 | 1 | 1 | 0 | 1 | 0 | 0 |
| | **6** | 0 | 15 | 0 | 0 | 15 | 0 | 0 | 15 | 0 | 0 | 15 | 0 | 0 | 15 | 0 | 1 | 14 | 0 | 7 | 7 | 0 | 5 | 2 | 0 | 1 | 1 | 0 | 1 | 0 | 0 |
| | **7** | 0 | 15 | 0 | 0 | 15 | 0 | 0 | 15 | 0 | 0 | 15 | 0 | 0 | 15 | 0 | 1 | 14 | 0 | 2 | 12 | 0 | 3 | 8 | 1 | 2 | 6 | 0 | 4 | 2 | 0 |
| **RSA-MW-500** | **8** | 0 | 15 | 0 | 0 | 15 | 0 | 1 | 14 | 0 | 0 | 14 | 0 | 0 | 14 | 0 | 0 | 14 | 0 | 4 | 10 | 0 | 2 | 8 | 0 | 2 | 6 | 0 | 2 | 4 | 0 |
| | **9** | 0 | 15 | 0 | 1 | 14 | 0 | 0 | 14 | 0 | 1 | 13 | 0 | 0 | 13 | 0 | 0 | 13 | 0 | 1 | 12 | 0 | 2 | 10 | 0 | 4 | 6 | 0 | 4 | 2 | 0 |
| | **7** | 0 | 15 | 0 | 0 | 15 | 0 | 0 | 15 | 0 | 0 | 15 | 0 | 0 | 15 | 0 | 1 | 13 | 1 | 0 | 12 | 1 | 0 | 12 | 0 | 0 | 12 | 0 | 4 | 8 | 0 |
| **RSA-MW-2343** | **8** | 0 | 15 | 0 | 0 | 15 | 0 | 0 | 15 | 0 | 1 | 14 | 0 | 0 | 14 | 0 | 4 | 9 | 1 | 0 | 9 | 0 | 1 | 8 | 0 | 3 | 5 | 0 | 1 | 4 | 0 |
| | **9** | 0 | 15 | 0 | 1 | 14 | 0 | 0 | 14 | 0 | 1 | 13 | 0 | 0 | 13 | 0 | 2 | 11 | 0 | 0 | 11 | 0 | 0 | 11 | 0 | 1 | 10 | 0 | 4 | 6 | 0 |
| | **7** | 0 | 15 | 0 | 0 | 15 | 0 | 0 | 15 | 0 | 0 | 15 | 0 | 2 | 13 | 0 | 0 | 13 | 0 | 4 | 9 | 0 | 0 | 9 | 0 | 0 | 9 | 0 | 4 | 5 | 0 |
| **RSA-MW-3980** | **8** | 0 | 15 | 0 | 1 | 14 | 0 | 0 | 14 | 0 | 0 | 14 | 0 | 0 | 14 | 0 | 1 | 13 | 0 | 6 | 7 | 0 | 0 | 7 | 0 | 2 | 5 | 0 | 2 | 3 | 0 |
| | **9** | 0 | 15 | 0 | 1 | 14 | 0 | 0 | 14 | 0 | 1 | 13 | 0 | 2 | 11 | 0 | 0 | 11 | 0 | 1 | 10 | 0 | 2 | 7 | 1 | 2 | 5 | 0 | 1 | 4 | 0 |
| | **7** | 0 | 15 | 0 | 0 | 15 | 0 | 1 | 14 | 0 | 0 | 14 | 0 | 1 | 13 | 0 | 0 | 12 | 1 | 0 | 10 | 2 | 3 | 6 | 1 | 1 | 5 | 0 | 0 | 5 | 0 |
| **RSA-MW-6610** | **8** | 0 | 15 | 0 | 0 | 15 | 0 | 0 | 15 | 0 | 0 | 15 | 0 | 0 | 15 | 0 | 1 | 13 | 1 | 0 | 12 | 1 | 2 | 10 | 0 | 2 | 7 | 1 | 4 | 3 | 0 |

| | | | | | | | | | | | | | | | | | | | | | | | | | | | | | | | |
|---|---|---|---|---|---|---|---|---|---|---|---|---|---|---|---|---|---|---|---|---|---|---|---|---|---|---|---|---|---|---|---|
| | **9** | 0 | 15 | 0 | 0 | 15 | 0 | 0 | 15 | 0 | 1 | 14 | 0 | 1 | 13 | 0 | 0 | 13 | 0 | 3 | 10 | 0 | 0 | 10 | 0 | 1 | 9 | 0 | 4 | 5 | 0 |
| **RSA-MW-4243** | **7** | 0 | 15 | 0 | 0 | 15 | 0 | 0 | 15 | 0 | 1 | 14 | 0 | 1 | 13 | 0 | 2 | 11 | 0 | 0 | 11 | 0 | 0 | 11 | 0 | 2 | 9 | 0 | 6 | 3 | 0 |
| | **8** | 0 | 15 | 0 | 0 | 15 | 0 | 0 | 15 | 0 | 0 | 15 | 0 | 0 | 15 | 0 | 0 | 15 | 0 | 1 | 14 | 0 | 3 | 11 | 0 | 1 | 10 | 0 | 1 | 9 | 0 |
| | **9** | 0 | 15 | 0 | 1 | 14 | 0 | 0 | 14 | 0 | 0 | 14 | 0 | 0 | 14 | 0 | 1 | 13 | 0 | 2 | 11 | 0 | 2 | 9 | 0 | 3 | 6 | 0 | 3 | 3 | 0 |
| **RSA-MW-2399** | **7** | 0 | 15 | 0 | 0 | 15 | 0 | 0 | 15 | 0 | 0 | 15 | 0 | 1 | 14 | 0 | 2 | 12 | 0 | 0 | 12 | 0 | 7 | 5 | 0 | 5 | 0 | 0 | 0 | 0 | 0 |
| | **8** | 0 | 15 | 0 | 1 | 14 | 0 | 0 | 14 | 0 | 0 | 14 | 0 | 1 | 13 | 0 | 1 | 12 | 0 | 0 | 12 | 0 | 5 | 7 | 0 | 4 | 3 | 0 | 3 | 0 | 0 |
| | **9** | 0 | 15 | 0 | 0 | 15 | 0 | 0 | 15 | 0 | 0 | 15 | 0 | 0 | 15 | 0 | 1 | 14 | 0 | 3 | 11 | 0 | 2 | 9 | 0 | 2 | 7 | 0 | 4 | 3 | 0 |

| | |
|---|---|
| **Dead** | **D** |
| **Alive** | **A** |
| **Censored** | **C** |
